# Emergent Freestanding Complex Oxide Membranes for Multifunctional Applications

**DOI:** 10.1002/adma.202521388

**Published:** 2026-02-12

**Authors:** Baowen Li, Yanran Liu, Jinjin Liu, Josephine Si Yu See, Kaijian Xing, Dong‐Chen Qi, Xiao Renshaw Wang

**Affiliations:** ^1^ Division of Physics and Applied Physics School of Physical and Mathematical Sciences Nanyang Technological University Singapore Singapore; ^2^ Macau University of Science and Technology Zhuhai MUST Science and Technology Research Institute Zhuhai China; ^3^ School of Physics and Astronomy Monash University Clayton Victoria Australia; ^4^ Center for Materials Science School of Chemistry and Physics Queensland University of Technology Brisbane Queensland Australia; ^5^ School of Electrical and Electronic Engineering Nanyang Technological University Singapore Singapore

**Keywords:** complex oxide, freestanding oxide membranes, two‐dimensional materials, van der Waals heterostructures, van der Waals integration

## Abstract

Correlated complex oxides feature tightly coupled charge, spin, orbital, and lattice degrees of freedom, which give rise to rich correlated behavior. Freestanding oxide membranes render these materials into tunable quasi‐2D platforms that enable multifunctional and reconfigurable devices. This Review surveys recent advances in the research of freestanding oxide membranes, highlighting their coupled correlated properties. We focus on three development pathways: (i) strain‐free membranes, (ii) strained membranes, and (iii) van der Waals‐integrated heterostructures. This organization begins with the intrinsic properties of oxide membranes, then examines mechanical tuning, heterogeneous integration, and multiphysics coupling to provide a comprehensive account of the field's development. Finally, we evaluate practical challenges, including high‐quality surfaces, robust multiphysics coupling, wafer‐scale transfer, and silicon‐compatible heterogeneous integration. Addressing these challenges will enable scalable, high‐yield manufacturing and expand the design space for oxide‐based architectures, thereby accelerating the transition from laboratory demonstrations to industry‐ready systems.

## Graphical abstract

This review surveys freestanding oxide membranes and covers fabrication and three pathways for studies and devices: strain‐free and strained membranes, and van der Waals‐integrated heterostructures. We show how coupled oxide responses map onto these routes and cross‐couple to expand behaviors. We identify barriers to scalable multifunctional devices and outline considerations for membrane quality, heterostructure construction, Si‐compatible processing, and wafer‐scale handling.

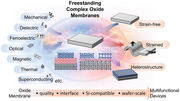

## 1 Introduction

Electronic applications, including modern optoelectronics, spintronics, and energy‐efficient electronics, underpin nearly every facet of modern life, from power generation and satellite navigation to voice‐controlled lighting and handheld devices [1]. Yet as the functional demands on materials diversify, there is a growing need for systems that exhibit richer, more unconventional electronic behavior. Strongly correlated electron materials meet this challenge by introducing intense electron–electron and electron–lattice interactions that reshape electronic states and energetics. These materials span complex metal oxides, heavy‐fermion and Kondo lattices, and low‐dimensional or topological platforms. Among various correlated systems, complex metal oxides stand out for their structural flexibility, complex orbital degrees of freedom, strong correlations, rich phase diagrams, field tunability, and advanced synthesis maturity, making them ideal candidates for multifunctional electronic systems [2].

Complex metal oxides host a wealth of correlated phenomena, including high‐temperature superconductivity [3, 4], Mott metal–insulator transitions [5], ferromagnetism [6, 7, 8], ferroelectricity [9], orbital order [10], and even electronic vortex states [11]. These responses are highly sensitive to atomic‐scale structure such as layer stacking, cation stoichiometry, and surface termination, making epitaxial deposition techniques, such as molecular beam epitaxy (MBE) and pulsed‐laser deposition (PLD), widely used routes for their synthesis [12, 13]. With unit‐cell precision and precisely controlled fluxes, epitaxial methods assemble films through adsorption and surface diffusion on single‐crystal templates, which keeps the film elastically clamped to the substrate and locking in both epitaxial misfit and thermal stresses. Once the critical thickness is exceeded, strain relaxation occurs through dislocations that degrade carrier mobility and increase leakage [14]. In addition, substrate clamping modifies domain configurations in ferroic oxides, suppresses switchability, and obscures intrinsic size‐ and strain‐dependent responses. Moreover, strong interfacial coupling promotes efficient but difficult‐to‐control phonon transmission, constraining thermal management in dense device architectures [15]. Together, these conditions impose lattice‐matching constraints, electromechanical rigidity, and difficult‐to‐engineer thermal pathways that limit device performance and obscure their intrinsic physics.

**FIGURE 1:**
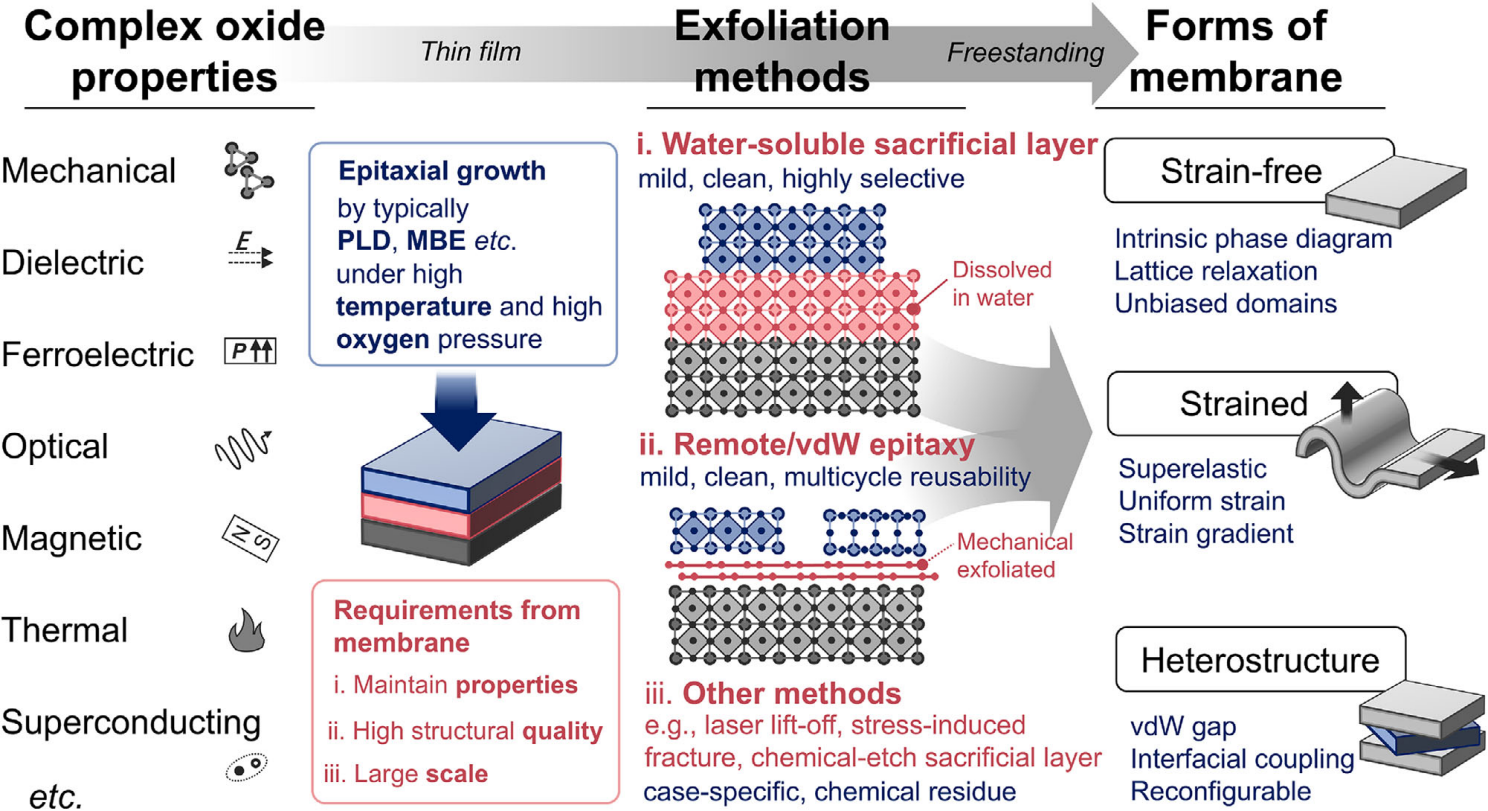
Overview of freestanding oxide routes. Complex oxides exhibit diverse ferroic, electronic, optical, and thermal properties, yet epitaxial thin films remain mechanically clamped by growth substrates. To obtain freestanding membranes that retain intrinsic properties, high structural quality, and scalability, several lift‐off strategies have been developed, including water‐soluble sacrificial layers, remote or vdW epitaxy, and other physical or chemical lift‐off routes with varying cleanliness and reusability. The resulting membranes further evolve along three complementary pathways: strain‐free membranes, strained membranes, and vdW‐assembled heterostructures, revealing intrinsic phase behavior, tunable strain states, and reconfigurable interfacial couplings.

Liberating epitaxial oxides from substrate clamping not only overcomes these limitations but also expands the available degrees of freedom for materials control, facilitates direct integration with industrial silicon (Si) and other platforms such as polymers, two‐dimensional (2D) materials, and provides additional handles for multiphysics modulation. To realize this, several lift‐off routes have been developed, including laser lift‐off [16], stress‐induced fracture [17, 18], sacrificial‐layer strategies [19, 20, 21, 22, 23, 24, 25], and remote/van der Waals (vdW) epitaxy [26, 27], among others. Each route serves distinct application scenarios and has delivered notable advances. Among various lift‐off routes, sacrificial‐layer strategies, especially those employing water‐soluble layers, and remote or vdW epitaxy have emerged as the most prominent in recent years, owing to their practicality, generality, and scalability. These approaches enable oxide membranes that operate as genuine quasi‐2D systems (Figure 1).

These correlated functionalities evolve in distinctive ways once complex oxides are made freestanding. The primary change is the release of lattice degrees of freedom from substrate‐imposed mechanical boundary conditions, which provides the central basis for organizing how different functionalities change. Mechanical compliance, dielectric permittivity, and ferroelectric order offer particularly direct readouts of declamping because they couple directly to lattice distortions, strain gradients, and polar instabilities. By contrast, optical, magnetic, superconducting, and thermal behaviors are often tuned through lattice‐mediated modifications of electronic bands, exchange and spin–orbit interactions, phonon spectra, or collective excitations. This separation between primary lattice‐coupled responses and lattice‐mediated multiphysics responses provides a practical framework for analyzing how diverse functionalities evolve. A multiphysics perspective enables cross‐validation of underlying mechanisms and lays the groundwork for coupling different functionalities. Accordingly, this Review covers a broad range of properties rather than focusing on a single property class, and discusses them within a unified framework.

More importantly, their study and utilization can be organized around three complementary pathways. First, releasing epitaxial films from substrate clamping restores a nearly strain‐free state that most faithfully reflects the intrinsic properties of the material, providing a foundational baseline for subsequent investigations. Second, whereas epitaxial films exhibit properties that superimpose intrinsic responses with misfit‐strain effects, freestanding membranes laminated onto flexible substrates enable continuous, programmable strain control. Beyond elucidating strain–property couplings, this pathway directly supports applications in flexible electronics. As materials approach the 2D or quasi‐2D limit, surface and interfacial contributions become increasingly significant. vdW‐assembled heterostructures, stacking homo‐ or hetero‐materials, including oxides and layered 2D crystals, allow the combination and tuning of parent properties and access to emergent interfacial phenomena, thereby greatly expanding the design space and functional degrees of freedom. Consequently, oxide heterostructure engineering forms the third pathway (Figure 1).

**FIGURE 2:**
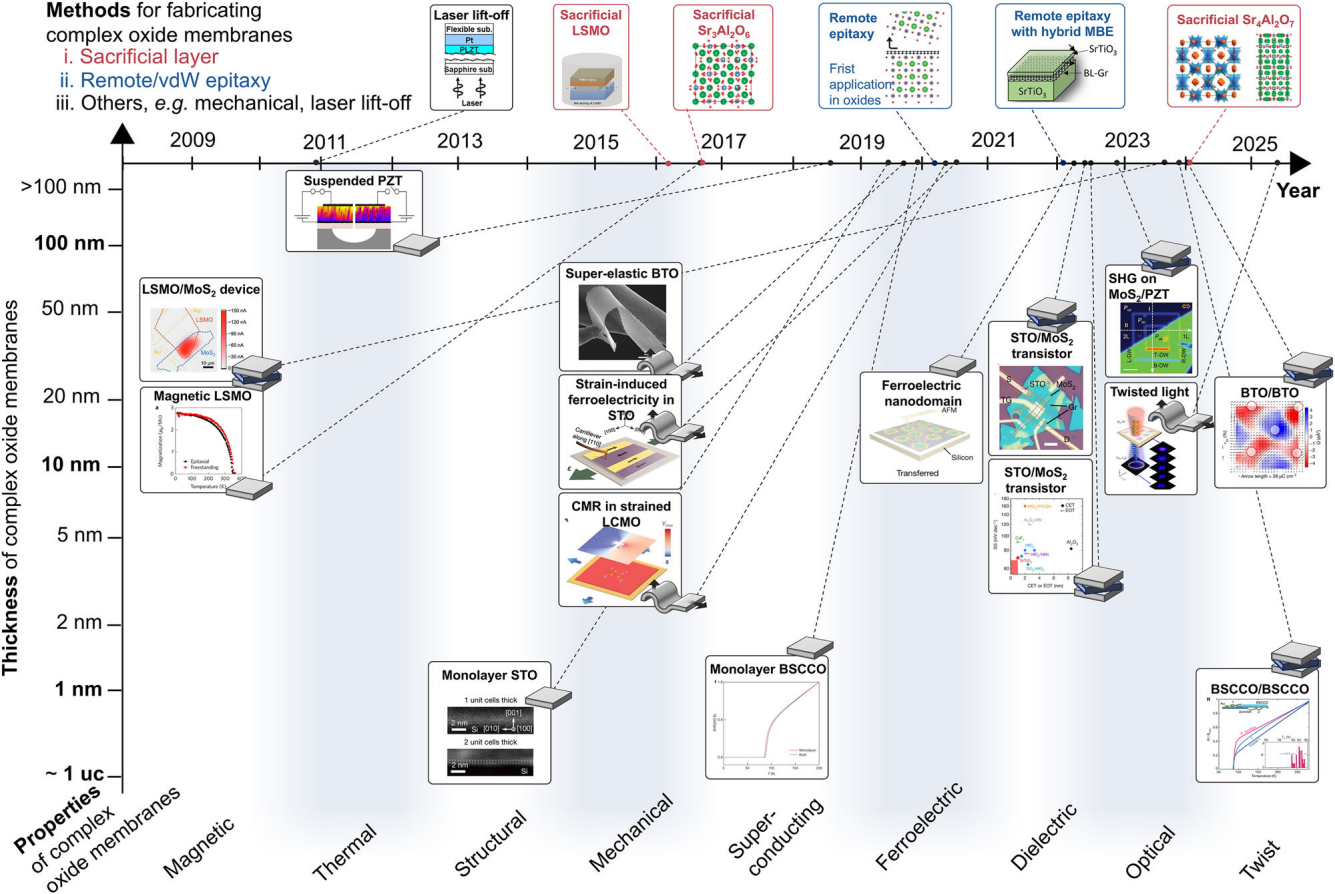
Expanding physical phenomena in freestanding complex oxides. As lift‐off techniques advance, freestanding complex oxides now encompass a broad spectrum of physical effects and functionalities. Methods for fabricating complex oxide membranes: laser lift‐off [16] (Copyright 2010, AIP Publishing), chemical etching by sacrificial layer (LSMO [19], Sr_3_Al_2_O_6_ [20, 21] (Copyright 2016, Springer), Sr_4_Al_2_O_7_ [21, 22] (Copyright 2024, The American Association for the Advancement of Science), remote epitaxy [37] (Copyright 2020, Springer) and remote epitaxy with hybrid MBE [38] (Copyright 2022, The American Association for the Advancement of Science). Representative material systems and device platforms: LSMO/MoS_2_ device [39] (Copyright 2023, Wiley), STO/MoS_2_ transistor [40, 41] (Copyright 2022, Springer), magnetic LSMO [20] (Copyright 2016, Springer), suspended PZT [42] (Copyright 2018, American Chemical Society), monolayer STO [43] (Copyright 2019, Springer), superelastic BTO [44] (Copyright 2019, The American Association for the Advancement of Science), BTO/BTO [45], monolayer BSCCO [46] (Copyright 2019, Springer), twisted BSCCO/BSCCO [47] (Copyright 2023, The American Association for the Advancement of Science). Representative effects and demonstrations: strain‐induced ferroelectricity in STO [48], CMR in strained LCMO [49] (Copyright 2018, American Chemical Society), ferroelectric nanodomain [50] (Copyright 2022, Springer), SHG on MoS_2_/PZT [51], twisted light in dome‐shaped BTO [52] (Copyright 2025, Springer).

As research on freestanding oxides continues to progress rapidly, recent reviews have offered systematic and informative overviews that mainly emphasize fabrication challenges [28], device design [29, 30, 31, 32, 33], or representative material case studies [34, 35, 36]. Nevertheless, it remains useful to adopt a complementary framework that starts from the wide range of oxide functionalities and follows how these responses evolve along three pathways. This framework begins with the strain‐free membrane as a foundational baseline, which helps establish an intrinsic physical picture before moving to deliberately strained membranes and heterostructure integration. This organization provides a more complete picture of freestanding oxides and offers clearer guidance for designing multiphysics‐coupled states and multifunctional devices. Accordingly, this Review is organized as follows. Section 2 introduces the two widely adopted fabrication routes (water‐soluble sacrificial layers and remote or vdW epitaxy) and discusses scalable pathways to achieve large‐area, crack‐free membranes. Sections 3, 4, 5 trace magnetic, thermal, structural, mechanical, superconducting, ferroelectric, dielectric, and optical responses from the strain‐free baseline, through strain modulation, to vdW‐assembled heterostructures and multiphysics coupling (Figure 2). We also place particular emphasis on interfacial mechanisms in Section 5. Finally, we outline the main challenges for oxide research and industrial application, and propose next steps to broaden interfacial design, improve control over structure–property relations, and move toward multifunctional devices with coupled multiphysics.

## 2 Fabrication of Freestanding Oxides

Growth defines a material's baseline properties. In complex oxide thin films, crystalline quality serves as the cornerstone of device performance and stability. Epitaxial growth techniques—such as molecular beam epitaxy (MBE), hybrid‐MBE, pulsed laser deposition (PLD), and magnetron sputtering (MS)—enable the creation of sharp interfaces with precise compositional control. Consequently, epitaxy remains the primary route for obtaining device‐grade correlated oxides. The translation of these advances is still bottlenecked by reliance on costly single‐crystal substrates and the challenge of releasing high‐quality films without inducing damage.

To realize freestanding oxides, early efforts focused on material‐specific, scenario‐dependent routes such as laser lift‐off [16], controlled surface cracking of the substrate [17, 18]. While these approaches have advanced our understanding of freestanding oxides, they lack generality and are difficult to extend across broad oxide families. To address this limitation, sacrificial‐layer strategies insert a selectively etchable or dissolvable interlayer between the substrate and the target oxide, enabling separation through chemical processing [19]. Although more general than material‐specific routes, this approach still faces several challenges: limited selectivity between the sacrificial layer and the oxide, chemical residues and contamination, and associated constraints on device integration. The adoption of water‐soluble sacrificial layers addresses much of this bottleneck, enabling a mild, highly selective, and clean lift‐off process. In parallel, remote or vdW epitaxy covers the substrate with few‐layer vdW materials so that the substrate's periodic potential partially penetrates, or in the vdW‐epitaxy case, the 2D layer itself provides the crystallographic registry, to guide epitaxy while introducing a weakly bonded interface, thereby permitting clean, mechanical lift‐off and transfer [27, 38]. Given the diversity of available methods, we summarize the commonly used approaches and their key trade‐offs in Table 1. For methodological details, we direct interested readers to recent method‐focused reviews that provide protocols and comparative discussions [30, 36, 53, 54, 55, 56]. Here, we focus on the two most broadly scalable approaches: water‐soluble sacrificial‐layer strategies and remote or vdW epitaxy.

**TABLE 1:** Fabrication and transfer methods for freestanding oxide membranes.

Exfoliation methods	Principle	Key advantages	Major limitations	Typical materials	Transfer methods
Laser lift‐off	Pulsed laser ablation at the substrate‐film interface triggers thermal decomposition, facilitating the exfoliation process	High efficiency, low cost, and industrial viability; Contactless and residue‐free; Compatibility with etch‐resistant materials	Thermal stress‐induced micro‐fractures; Interfacial material damage; Requirement for a transparent substrate	PZT/sapphire [78, 79]	Compatible with both dry and wet transfer Dry method: The target thin film is first picked up by a solid Thermal release tape (e.g., PDMS stamp) and subsequently transferred to the final substrate via thermal activation Wet method: The target thin film is first coated by polymer support or metallic handle (e.g., PPC, Ni layer). After being transferred to the target substrate, the support layer will be dissolved in a suitable solvent (e.g., acetone) to expose the pristine oxide film
Mechanical Exfoliation/Controlled spalling	Self‐controlled spalling from substrates induced via interfacial dislocations or external auxiliary layers	Large‐scale and high efficiency Room‐temperature operation without thermal damage; No Sacrificial Layer required	Interfacial roughness; Residual stress‐induced bowing; Substrate damage	LAO/STO [17, 18]	
vdW epitaxy	The target thin films undergo direct nucleation and growth on smooth, dangling‐bond‐free substrate surfaces, such as Mica, resulting in an interface governed solely by weak vdW interactions that facilitate highly facile exfoliation	Permitting the growth of single‐crystalline films under a large lattice mismatch; Superior flexibility and transparency, which can be directly integrated into flexible devices	Co‐delamination of mica substrates, leaving residues; Inhomogeneous nucleation due to a lack of covalent bonding, impacting film crystallinity	STO/F‐mica [75] SRO‐mica [80]	
Remote epitaxy	Nucleation of the target thin film is directly guided by the substrate potential field through ultrathin 2D materials	Substrate recycling for cost reduction; Low interfacial adhesion, enabling facile exfoliation	Limited to highly polar substrates; Stringent growth conditions; Delamination failure and polycrystal growth due to pinhole‐mediated nucleation	STO/graphene/STO [37] BTO/graphene/STO [26, 69]	
Water‐soluble sacrificial layer‐assisted exfoliation	Selective dissolution of the sacrificial interlayer between target films and substrates	Simple and safe operation Low cost; Reusability of the substrate; Acid‐free, environmentally friendly; Tunable lattice constant of the sacrificial layer	Restricted to water‐stable oxide films; Degradation of film quality due to lattice mismatch with the sacrificial layer; Lack of robust protocols for scalable transfer	STO/SAO/STO [20] LSMO/SAO/STO [20] SRO/SAO_T_/STO [21] STO/SAOT/STO [21, 22] STO/SVO/STO [64]	Compatible with wet transfer Support‐layer‐assisted transfer: The oxide film was transferred using a pre‐coated polymer support, which was removed after sacrificial layer dissolution and placement on the target substrate Direct scooping transfer: Upon sacrificial layer dissolution, the self‐supporting membrane was directly scooped onto the target substrate
Other chemical etching, sacrificial layer‐assisted exfoliation		Simple operation; Reusability of the substrate	Hazardous chemical waste, environmentally unfriendly Solvent residue risk	PZT/LSMO/STO(KI+HCl) [81] BFO/SMO/STO(KI+HCl) [23]	

### 2.1 Water‐Soluble Sacrificial‐Layer Strategies

Water is an ideal etchant for sacrificial layers: it is benign, inexpensive, and highly selective. A key advance came in 2016, when Lu et al. introduced epitaxial Sr_3_Al_2_O_6_ (SAO_C_) as a water‐soluble buffer to realize transferable, single‐crystal perovskite membranes [20]. SAO_C_ is cubic (*a* ≈ 15.844 Å) and forms a 4:1 coincidence with SrTiO_3_ (STO) (4 × *a*_STO_ ≈ 15.620 Å), enabling high‐fidelity growth while preserving a clean, water‐etchable lift‐off pathway. Beyond the parent compound, *A*‐site doping (Sr to Ca/Ba) and choice of coincidence ratios broaden the effective matching window to cover a wide range of perovskite pseudocubic lattices. This flexibility extends the method to diverse ABO_3_ films and heterostructures [57, 58, 59]. In 2024, two independent reports established “supertetragonal” Sr_4_Al_2_O_7_ (SAO_T_) as a second‐generation, water‐soluble sacrificial layer [21, 22]. The low‐symmetry lattice of SAO_T_ better accommodates epitaxial strain and delivers high lattice compatibility across ≈ 3.85–4.04 Å *AB*O_3_ films. At the chemical level, its Al–O framework comprises more discrete polyhedral units, which hydrolyze readily. In water, SAO_T_ dissolves roughly an order of magnitude faster than SAO_C_, which enables continuous, millimeter‐scale freestanding membranes and improves process throughput.

The discovery of SAO provided a simple, broadly applicable sacrificial‐layer route to freestanding perovskite oxide membranes, and it remains the most widely used preparation strategy. This strategy enables high‐quality single‐crystal perovskite films, including STO, BiFeO_3_ (BFO), Sr‐doped LaMnO_3_ (LSMO), and BaTiO_3_ (BTO) [58, 60, 61, 62]. A persistent caveat, however, is cation interdiffusion at growth temperatures: diffusion along lattice defects can generate chemically modified interfacial “dead layers” that degrade functional responses (for example, in LSMO/SAO stacks) [60, 63]. Ultrathin STO interlayers can partially mitigate interdiffusion, but they remain after lift‐off, which adds capacitance and introduces additional interfaces. This creates a clear trade‐off between interfacial chemistry control and device‐stack simplicity [60]. An alternative is water‐soluble SrVO_3_, which enables the transfer of nanometer‐thick STO onto technologically relevant platforms [64]; yet its slower dissolution kinetics (even at elevated temperature) limit throughput relative to SAO_C_/SAO_T_. Finally, because SAO is highly hygroscopic, direct coupling to aqueous or hydrolysis‐prone chemical solution deposition requires careful solvent selection and process control. Nonetheless, recent all‐solution processes show that controlling precursor hydrolysis can reconcile SAO with chemical solution deposition and deliver freestanding, single‐crystal BTO membranes [65].

### 2.2 Remote/vdW Epitaxy

Using atomically thin and dangling‐bond‐free 2D interlayers, including graphene, hexagonal boron nitride (hBN), and transition‐metal dichalcogenides (TMDs), enables epitaxy without forming a conventional covalent interface while preserving a layer‐transfer (exfoliable) lift‐off pathway. The mechanistic basis is the partial penetration of the substrate's periodic electrostatic potential through a monolayer (or bilayer) 2D sheet. Adatoms diffusing on the 2D layer sense an attenuated yet registry‐preserving “remote” potential and nucleate accordingly. This mechanism is known as remote epitaxy. The strength of lattice transparency depends on 2D thickness, substrate ionicity/polarity, and the electronic properties and defect density of the 2D layer.

Within oxide heteroepitaxy, graphene and MoS_2_ have emerged as the predominant 2D spacer layers, owing to their monolayer thickness which enables lattice transparency and integration versatility. High‐quality freestanding oxides have been realized on graphene platforms, including BTO, STO and Pb(Zr_1‐_ *_x_* Ti*_x_*)O_3_ (PZT) [26], and analogous integration on MoS_2_ has already produced STO and CoFe_2_O_4_ (CFO) [66]. Owing to the weak vdW coupling between graphene and the overgrown oxide, the film can be readily exfoliated from the substrate, achieving freestanding transfer.

Remote epitaxial growth of oxide thin films remains challenging. The key issue is structural incompatibility between fragile 2D interlayers and the oxide growth environment, which often involves high thermal budgets and energetic pulsed‐laser deposition flux. These conditions can damage the 2D template and narrow the practical remote‐epitaxy window. For graphene‐based approaches, the dominant failure modes during oxide growth are oxidation and bombardment by energetic species or plasma. These effects can be mitigated by lowering oxygen activity, introducing an Ar background to thermalize the plume, and reducing the kinetic‐energy flux through gentler ablation conditions such as a smaller laser spot, a larger target–substrate distance, and softer ablation. These measures help preserve graphene and sustain remote epitaxy. For MoS_2_ interlayers, the main constraint is thermal and chemical stability. Avoiding sulfur loss and oxidation requires a tighter growth window that uses moderate temperatures, controlled oxygen activity, and a gentle particle flux.

Beyond the stringent growth window, pinhole‐mediated nucleation is another practical challenge in remote epitaxy [67]. In this pathway, nucleation occurs through microscopic pinholes or defects in the 2D layer, which allows direct chemical bonding to the underlying substrate and is followed by lateral overgrowth. Whether this pinhole route dominates over true remote‐field‐driven nucleation remains actively debated.

Support for pinhole dominance includes reports of epitaxial single‐crystal growth on nonpolar or weakly polar substrates [68], where a penetrating substrate potential field should be negligible. Additional support comes from epitaxial growth on multilayer graphene [69, 70], where strong screening should further suppress remote‐field transmission. At the same time, multiple observations support the existence of remote epitaxy. Du et al. observed epitaxial domains rotated by 30° relative to the substrate in GdPtSb/graphene/Al_2_O_3_ [71]. Because pinhole‐mediated nucleation involves direct bonding to the substrate, it typically yields the substrate‐matched orientation. The rotated domains are therefore difficult to reconcile with a purely pinhole‐driven picture and have been viewed as a mechanistic fingerprint of remote epitaxy. Chang et al. [69] also used scanning transmission electron microscopy to identify continuous, intact graphene beneath isolated nanocrystalline nuclei, which supports nucleation without pervasive direct bonding through pinholes.

A prevailing view is that remote epitaxial nucleation and pinhole‐mediated nucleation coexist and compete during growth. Chang et al. [69] patterned graphene by EBL to emulate pinhole defects and then grew BTO, which enabled a systematic assessment of how both mechanisms influence single‐crystal film formation. Their results identify the number of 2D layers, the polarity of the underlying substrate, and the growth temperature as key variables that govern which prevails experimentally. Pinhole‐mediated nucleation is generally detrimental for remote epitaxy. Direct covalent bonding between the film and the substrate hinders nondestructive exfoliation and prevents reuse of expensive substrates, which undermines a major practical motivation for remote epitaxy. Pinhole‐driven lateral overgrowth can yield large‐area continuous single‐crystal films under specific conditions when defect distributions are uniform [72]. In typical cases, pinholes are randomly distributed. The resulting competition between remote and pinhole mechanisms introduces orientational conflicts that promote polycrystalline growth and degrade crystalline quality.

When the 2D spacer increases in thickness (from monolayer to multilayers), substrate fields are screened, and the film sees only the 2D template, i.e., growth transitions from remote‐epitaxy‐dominated (substrate potential partially penetrates a monolayer) to true vdW epitaxy governed by the 2D lattice. Compared with remote epitaxy, vdW epitaxy aligns to the 2D spacer lattice rather than the underlying substrate, inherently tolerating large lattice and symmetry mismatches without generating misfit dislocations. It therefore applies equally well to non‐ionic, low‐polarity, and even inert substrates. Moreover, because the weak 2D–overlayer interaction governs growth, modest defects in the 2D layer do not trigger the debated pinhole mechanism of remote epitaxy, mentioned above, and orientation design and substrate lift‐off are more direct. Beyond conventional 2D spacers, vdW epitaxy on mica has attracted wide interest, especially for flexible devices [73, 74]. Using this approach, Lu et al. synthesized single‐crystal STO and LSMO films on mica and verified their exceptional crystalline quality [75].

### 2.3 Transfer Strategies for Large‐Scale Freestanding Oxide Membranes

For freestanding oxide films obtained through lift‐off methods, the transfer step is equally critical to device fabrication. In particular, scalable manufacturing requires membrane integrity to be preserved during handling and process‐borne contamination to be avoided because it degrades interfaces and limits device performance. At present, a common transfer route for perovskite oxides uses a one‐step stamp process. A stamp with an adhesive layer and a support layer contacts the released film, lifts it from the growth substrate, and places it onto the target substrate or a 2D material. The adhesive is then removed via thermal release, mechanical peeling, or selective dissolution. Practical choices for the adhesive/support include polymethyl methacrylate (PMMA), polydimethylsiloxane (PDMS), and polyethylene terephthalate (PET) [20, 76]. While straightforward and broadly compatible, the one‐step method typically yields crack‐free areas from micrometers to at most the sub‐millimeter scale. To overcome this, a two‐step transfer was introduced in 2024 by An et al.: a compliant PDMS layer first cushions residual strain associated with lattice‐mismatch‐induced stress during lift‐off, minimizing fracture; the film is then retrieved from PDMS using polypropylene carbonate (PPC), which softens into a glassy state upon heating and enables a clean interface on flexible supports (e.g., Si wafers or PET) [77]. This route reproducibly delivers sub‐centimeter‐scale, intact oxide membranes.

## 3 Exploration on Strain‐Free Freestanding Complex Oxides

Freestanding correlated oxide membranes remove the epitaxial clamping imposed by growth substrates. We refer to this clamping‐free state as strain‐free. In this usage, “strain‐free” denotes a change in boundary conditions rather than a perfectly undeformed lattice, and local deformations or residual strain can still arise depending on geometry and boundary conditions. It is worth noting that strain‐free freestanding oxides may still be mounted on flexible carriers or other supports to facilitate handling and measurement. Such support is intended to retain the released, clamping‐free boundary condition and is not used to impose controlled mechanical strain. Any externally applied deformation discussed in this section serves as a mechanical probe for extracting intrinsic parameters such as elastic constants and bending stiffness. Studies that intentionally use deformation as a tuning variable to reshape ferroelectric behavior or other functional responses are discussed in Section 4 on strained membranes. The clamping effect is particularly pronounced at thicknesses of only a few unit cells (uc) to several nanometers, where substrate‐induced artifacts can dominate apparent size scaling and obscure intrinsic behavior. Release from the substrate restores structural degrees of freedom, allowing the lattice to deform under a range of boundary conditions. Crucially, such deformations couple directly to structure‐sensitive properties, including mechanical behavior and ferroelectric order, providing a broad platform for probing fundamental physics and establishing the basis for the flexible‐device strategies introduced in Section 4 to modulate diverse functionalities.

In this section, the structural stability and mechanical response of freestanding oxide membranes are first established (Sections 3.1 and 3.2), thereby defining how the lattice accommodates deformation once substrate clamping is removed. Dielectric and ferroelectric properties are then examined (Sections 3.3 and 3.4), as they often provide the most direct signatures of lattice relaxation. We finally turn to optical, magnetic, superconducting, and thermal functionalities (Sections 3.5–3.8), where the influence of freestanding conditions is more commonly manifested through lattice‐mediated modifications of electronic structure and collective excitations, rather than through a direct mechanical response. Taken together, the strain‐free freestanding geometry enables enhanced coupling among mechanical, electronic, and collective degrees of freedom, providing a foundation for multifunctional behavior in oxide membranes.

### 3.1 Structural Control on Strain‐Free Membranes

**FIGURE 3:**
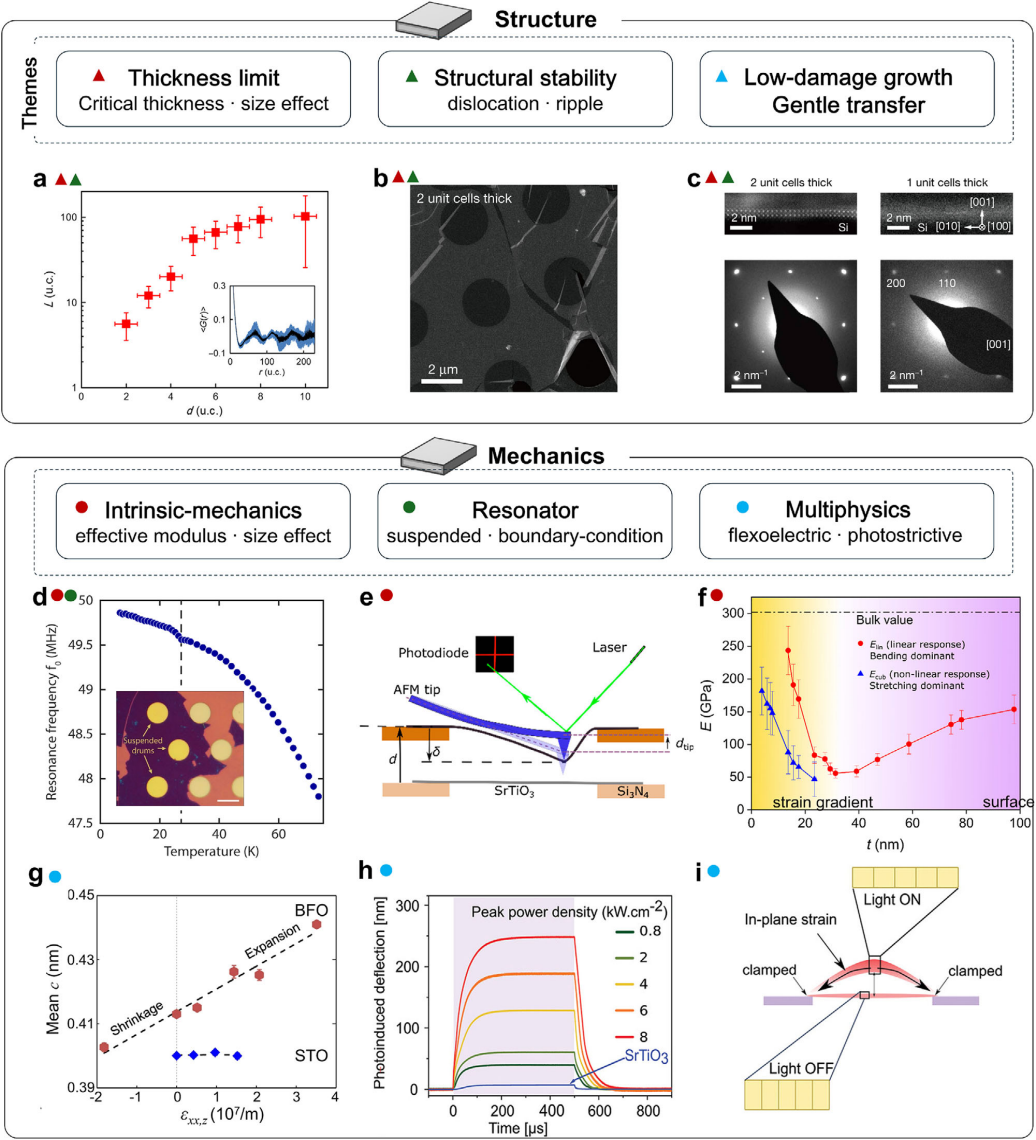
Structural and mechanical properties of strain‐free complex oxide membranes. (a) Thickness‐dependent crystalline coherence length (*L*) computed from the averaged spatial correlation function (*G*) derived from dark‐field TEM images of STO; Inset, raw correlation function from a 6‐uc membrane [82]. (b) Low‐magnification plan‐view high‐angle annular dark field (HAADF) image of a 2‐uc freestanding STO film. (c) Cross‐sectional HAADF (top) and selected area electron diffraction (SAED) (bottom) of ultrathin freestanding STO films with various uc thicknesses [43]. (d) Resonance frequency versus temperature. Inset: optical image of suspended 9‐uc SrRuO_3_ (SRO) drums [83]. Scale bar, 10 µm. (e) Measurement schematic of nanodrum deformation. (f) Thickness dependence of the effective Young's modulus for linear and cubic responses in STO membranes [84]. (g) Mean lattice spacing (*c*) versus in‐plane strain gradient in bent BFO and STO [85]. (h) Pulsed excitation of a BTO membrane; at 500 µs pulse width the out‐of‐plane deflection increases and saturates. (i) Schematic of the process resulting in actuation of the membranes [86]. Panels b and c reproduced with permission: Copyright 2019, Springer. Panels e and f reproduced with permission: Copyright 2021, American Chemical Society. Panels h and i reproduced with permission: Copyright 2021, Wiley.

In the chip era, ultrathin 2D films are a coveted goal. On substrates, correlated oxides can be grown with atomic‐layer precision. Once the substrate is removed, however, a prerequisite for any functional response is structural integrity. Can such ultrathin oxides remain crystalline and self‐supporting, as graphene does, and do new thickness‐driven size effects appear? To address this question, Hong et al. fabricated freestanding STO membranes (1–10 uc) from epitaxial films and found long‐range order persisting to ∼5 uc [82] (Figure 3a); below this, transmission electron microscopy (TEM) showed an exponential decline in coherence length and a sharp rise in dislocation density, signaling a critical thickness where order is lost, and dislocation pairs accommodate strain. This fragility was attributed to the substantial bond‐breaking energy released during lift‐off in ionic compounds. Subsequent work on MBE‐grown films, however, reported single‐crystal order down to a single‐unit‐cell, accompanied by pronounced roughening suggestive of dislocation‐free, ripple‐like stabilization [43] (Figure 3b,c). Lower‐energy growth and transfer routes, such as remote epitaxy, have been proposed, though their extension to a broader range of single‐unit‐cell oxide membranes is still evolving [37, 38]. Overall, crystallinity in ultrathin oxide membranes is highly process‐dependent; careful optimization of growth, lift‐off, and weakly bonded interfaces will define the window in which true monolayer perovskites are stable.

### 3.2 Mechanical Properties of Strain‐Free Membranes

Once crystallinity and structural stability are established, the most immediate consequence of releasing substrate clamping is the emergence of unconventional mechanical responses. Freestanding oxide membranes can sustain large strains and strain gradients. Their device‐level mechanical response is ultimately governed by their intrinsic elastic moduli, which are relevant to both mechanical and electronic applications. In 2020, Davidovikj et al. realized ultrathin STO and SrRuO_3_ (SRO) nanodrums on SiO_2_ holes and showed that structural transitions imprint directly onto the resonance spectrum; tracking temperature‐dependent frequencies and linewidths revealed a phase transition that modulates both built‐in strain and mechanical loss [83] (Figure 3d). Most strikingly, nanomechanical tests on freestanding STO uncovered a thickness anomaly: below ∼20 nm, the bending‐derived modulus exceeds the stretching modulus by roughly a factor of three and varies non‐monotonically with thickness [84] (Figure 3e,f). This behavior is attributed to the strain‐gradient contributions from flexoelectricity, which are geometrically amplified in the suspended membranes. In addition, trampoline resonators fabricated from bulk‐micromachined STO membranes broaden both the metrology toolset and the accessible sample geometries, are inherently scalable for wafer‐scale fabrication, and thus usefully complement drum‐type resonators [87]. These devices are compatible with wafer‐scale fabrication and complement drum‐type resonators. Adhesion and bonding at the oxide–substrate rim set the edge tension and determine hermeticity in pressure‐driven devices. Engineering the suspension geometry has therefore not only unlocked practical advances in sensing, but also exposed a materials challenge: achieving leak‐tight cavities. Addressing this, Lee et al. introduced an anneal‐induced self‐sealing at the oxide/SiO_2_ interface that boosts cavity hermeticity by up to four orders of magnitude (permeation time constants from ∼14 to ∼1.2 × 10^5^ s), thereby stabilizing pressure readout and long‐duration mechanical metrology [88].

The functional landscape of freestanding oxide membranes is remarkably diverse, with many properties poised to couple to or be controlled by deformation mechanics. Ferroelectricity is a prime example, given its stringent dependence on lattice geometry and symmetry breaking. For example, Cai et al. conducted atomic imaging of wrinkled ferroelectric BFO and non‐polar STO membranes using a wrinkle structure, revealing giant flexoelectric polarization [85]. Furthermore, an unusual bending‐expansion/shrinkage effect in the ferroelectric BFO membrane is exhibited and explained by flexo–piezo interplay (Figure 3g). This reframes bending rigidity as electromechanically renormalized, impacting how one infers “effective” modulus from flexural tests. Interestingly, Ganguly et al. recently demonstrated photostrictive actuation in suspended BTO nanodrums on SiN windows [86] (Figure 3h). Under microsecond optical pulses (0.5–500 µs), the drums exhibit center deflections up to ∼250 nm and in‐plane expansion up to ∼0.047%, with a clear temperature dependence. The response is attributed to photoexcited carriers partially screening the ferroelectric polarization, reducing tetragonality (out‐of‐plane lattice constant *c* contracts, and in‐plane lattice constant *a* expands). In an edge‐clamped, center‐free drum, this anisotropic lattice change is converted into large out‐of‐plane deflection (Figure 3i). These results showcase tightly coupled photo–ferroelectric–mechanical (with thermal contributions) multiphysics in correlated oxides, highlighting their potential for multiphysics‐coupled induced hybrid complex devices.

### 3.3 Dielectric Properties of Strain‐Free Membranes

Beyond mechanical compliance, lattice relaxation in freestanding membranes profoundly reshapes how atomic displacements couple to electric fields, making dielectric response a particularly sensitive probe of the strain‐free state. This focus on dielectric behavior naturally highlights perovskite oxides, where many celebrated functionalities stem from a soft and strongly coupled lattice. Among them, STO stands as a prototypical high‐*κ* dielectric, where the Ti^4+^ ions at the *B*‐site of the perovskite lattice occupy oxygen octahedra (TiO_6_) that are easily displaced under an external electric field. This soft‐mode polarizability endows bulk STO with an exceptionally large dielectric constant, approximately 300 at room temperature [89, 90]. When thinned to the freestanding regime, however, the dielectric response of STO degrades dramatically relative to its bulk counterpart [40, 91, 92, 93]. The suppression of permittivity stems from the combined influence of altered lattice dynamics at reduced dimensionality. This change stiffens polar phonons and quenches soft‐mode fluctuations. Structural imperfections such as oxygen vacancies, strain gradients, surface reconstructions, and interfacial disorder further suppress the response. These extrinsic perturbations collectively freeze dipolar fluctuations and lower the effective polarizability of the membrane. A widely invoked framework to quantify this degradation is the “dead‐layer” model, which posits the presence of an interfacial region with strongly suppressed dielectric response between the high‐*κ* film and its adjoining electrode or semiconductor [94, 95, 96, 97]. Within this model, the overall capacitance can be expressed as *t*/*ε_eff_* = *t*/*ε_bulk_* + *D*, where *t* is the thickness of STO, *ε_eff_* is the effective dielectric constant, *ε_bulk_* is the dielectric constant of bulk STO, and *D* is the coefficient introduced by the presence of the dead layer. Huang et al. directly verified this scaling using metal–insulator–metal (MIM) capacitors based on freestanding STO membranes supported on Pt‐coated Si substrates [41], with the capacitance determined as *C_eff_* = *Aε_0_ε_eff_*/*t*. Their results revealed that when the STO thickness falls below 26 nm, the capacitance‐equivalent thickness decreases to under 1 nm, an exceptional figure for suppressing quantum tunneling in advanced metal‐oxide‐semiconductor (MOS) transistors, thereby sustaining device scaling consistent with Moore's law. Complementary dual‐gate and Hall‐effect measurements by Yang et al. further confirmed the robust dielectric performance of vdW‐integrated STO layers [40, 41]. The same MIM architecture also enabled characterization of leakage and breakdown behavior and showed that freestanding STO films transferred onto Si exhibit leakage currents (less than 10^−4^ A·cm^−2^) far below the low‐power limit (10^−2^ A cm^−2^) specified by the International Roadmap for Devices and Systems (IRDS) [98] and demonstrate breakdown fields exceeding those of most conventional high‐*κ* dielectrics.

### 3.4 Ferroelectric Properties of Strain‐Free Membranes

**FIGURE 4:**
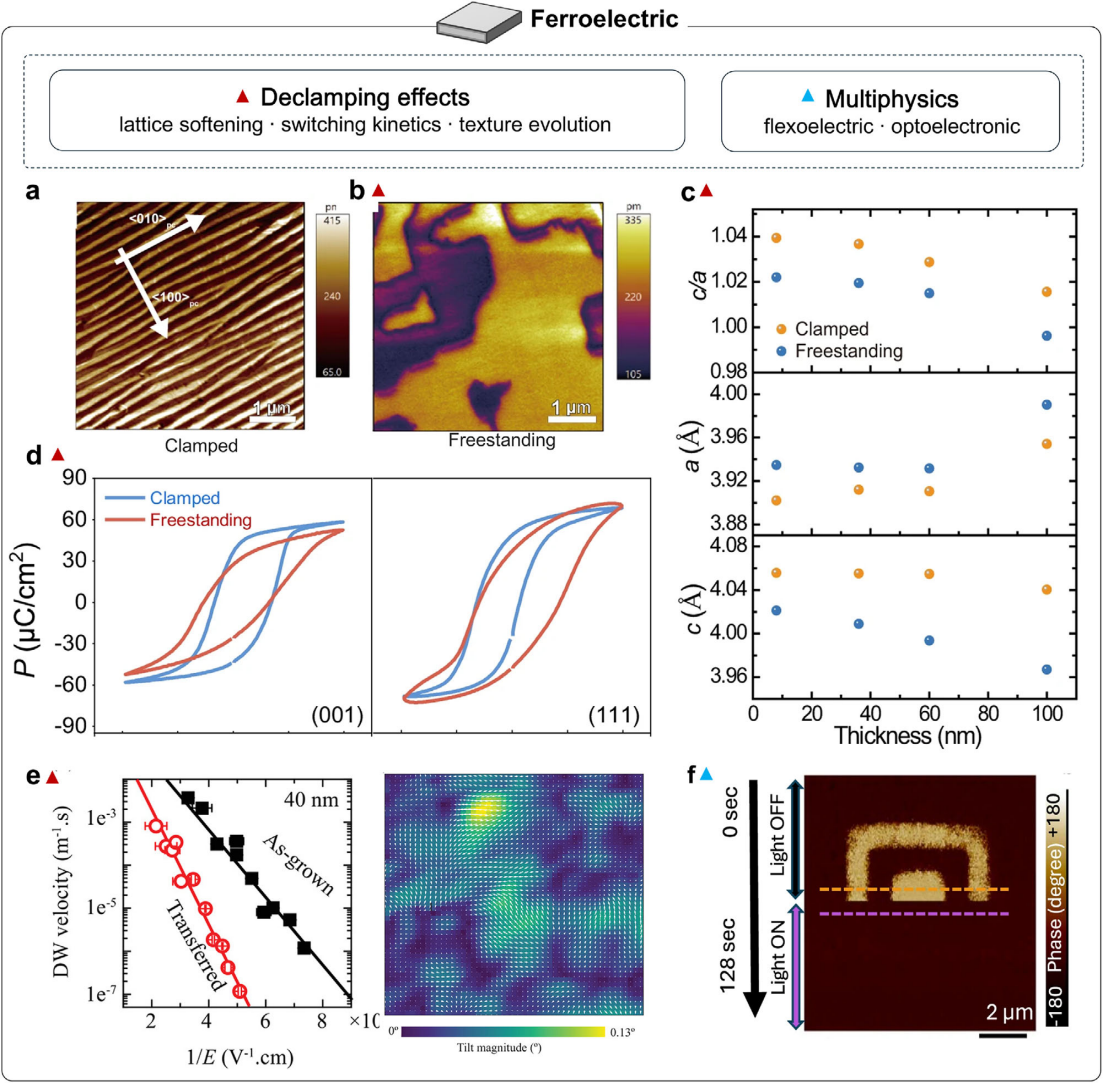
Ferroelectric properties of strain‐free oxide membranes. (a) Out‐of‐plane lattice constant (*c*) and in‐plane lattice constant (*a*), and the *c*/*a* ratio of BFO films and freestanding membranes as a function of thickness; membranes show decreased *c*, increased *a*, and reduced *c*/*a*. (b) In‐plane PFM‐amplitude image of a 100 nm BFO film. (c) In‐plane piezoelectric force microscopy (PFM)‐amplitude image of a freestanding BFO membrane [99]. (d) Comparison of polarization hysteresis (*P*‐*E*) loops for clamped and freestanding films [100]. (e) Domain‐wall velocities in 40 nm as‐grown and transferred films plotted versus 1/*E*, and crystallographic‐tilt map over a 1.5 × 1.5 µm^2^ region of transferred PZT; arrows indicate local tilt directions [81]. (f) Phase images acquired in the dark (top) and under illumination (bottom), with yellow and purple line scans corresponding to dark and illuminated conditions [102]. Panel e reproduced with permission: Copyright 2019, Wiley.

Building on dielectric response, which reflects the linear susceptibility of polar lattice distortions to an external electric field, ferroelectricity represents a further step in which such distortions stabilize into a spontaneous and switchable polarization accompanied by domain formation. In perovskite oxides, both dielectric and ferroelectric behaviors originate from polar lattice instabilities, but ferroelectric order amplifies this coupling by breaking inversion symmetry and introducing nonlinear switching dynamics. This enhanced lattice–electric‐field coupling renders ferroelectricity particularly sensitive to substrate clamping. In conventional epitaxial films, lattice matching to the substrate imposes mechanical constraints that restrict lattice relaxation and reshape domain configurations and switching energetics. Freed from these constraints, freestanding ferroelectric oxides offer a compelling platform to reveal intrinsic behavior and enable new device physics. Establishing this intrinsic ferroelectric picture also provides a clear baseline for later heterostructure devices, where polarization screening, domain stability, and switching thresholds directly shape device behavior. Addressing this point, Shi et al. compared freestanding BFO membranes transferred onto Si/Pt substrates with substrate‐clamped epitaxial films to isolate the role of lattice dynamics in ferroelectric switching [99] (Figure 4a–c). They observed a clear evolution of domain textures from ∼100 nm spaced striped 71° ferroelastic domains in clamped films to expansive 180° domains, with characteristic sizes of tens of micrometers, in freestanding membranes. Removing the mechanical clamp reduced the switching voltage by ≈40% and increased the switching speed by ≈60%. These results highlight a dynamic clamping process during switching that couples strain with ferroelectric and ferrodistortive order parameters, thereby setting the energetics and kinetics of polarization reversal. In addition, PZT undergoes a tetragonal‐to‐monoclinic transition after transfer onto a PDMS polymer substrate [100] (Figure 4d). The intrinsic high‐field permittivity (*ε*_i_) rises by ∼80%, while low‐field response shows amplified extrinsic and domain‐wall contributions, signatures of lattice softening, and enhanced polarization‐rotation capability. Additionally, Bakaul et al. (2019) showed that releasing single‐crystal PZT from its epitaxial growth substrate and transferring it onto an LSMO underlayer slows ferroelectric domain‐wall motion by three orders of magnitude compared with clamped epitaxial films [81]. This trend contrasts with the expectation that reduced mechanical constraint should accelerate domain‐wall motion (Figure 4e). They attributed the slowdown to mesoscopic surface ripples that emerge once clamping is removed. The associated strain gradients generate flexoelectric fields, reshaping the local energy landscape and raising the effective barrier for domain‐wall propagation, thereby suppressing its velocity. Although freestanding oxides are liberated from substrate clamping, they also lose substrate screening and become more sensitive to the ambient. To probe this, in situ scanning transmission electron microscopy (STEM) tracked domain configurations in freestanding BTO under controlled chemistry (vacuum versus oxygen‐rich) and temperature ramps [101]. The phase window and domain topology proved exceptionally environment‐sensitive. For example, an *a*–*c* to *a*–*a* low‐temperature reconstruction emerged around 60°C, and oxygen‐rich conditions suppressed domain formation. These behaviors are consistent with the loss of the bottom electrode: once the film is lifted off, surface charges and the surrounding atmosphere dominate depolarization screening, reshaping the domain landscape. Recently, Pal et al. demonstrated sub‐second (<500 ms) optical switching of polarization in freestanding BaTiO_3_ (BTO) membranes supported on a conducting ITO base. This switching simultaneously modulates the device resistance and enables optically controlled computing [102] (Figure 4f). They further showed that lift‐off from substrate clamping accelerates domain kinetics; combined with strain engineering, this affords full optical programmability of ferroelectric polarization. Overall, removing substrate clamping redefines the mechanical and electrical boundary conditions of ferroelectrics. This declamping opens a much broader parameter space for tuning polarization, domains, and coupled functionalities at lower activation thresholds.

### 3.5 Optical Properties of Strain‐Free Membranes

**FIGURE 5:**
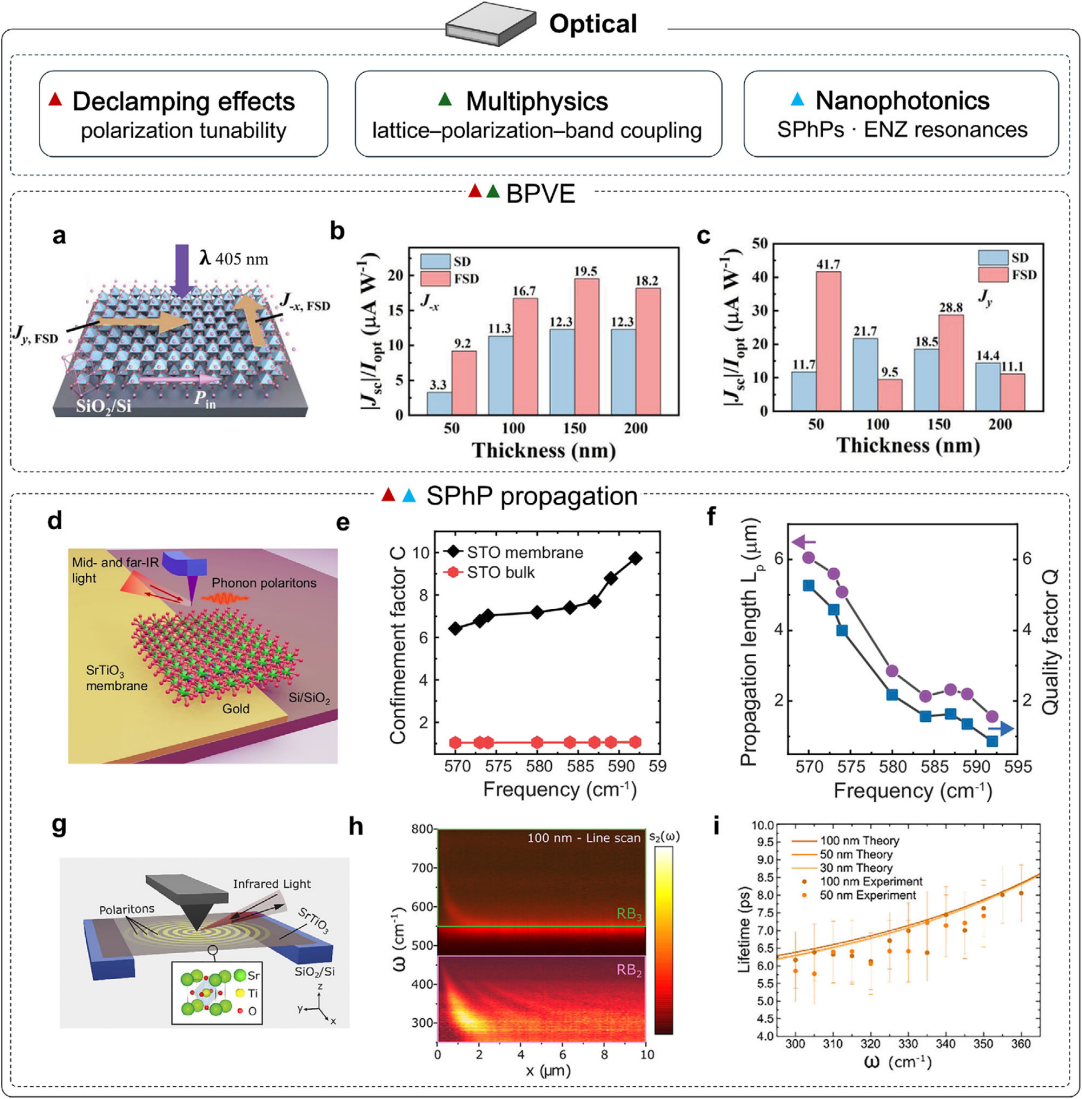
Optical properties of strain‐free complex oxide membranes. (a) Configuration for bulk photovoltaic effect (BPVE) measurements on freestanding single‐domain (FSD) samples. Pink arrows (*P*_in_) indicate the in‐plane polarization components; yellow arrows mark the positive *J*_sc_ direction. (b,c) Statistical comparison of |*J*_sc_|/*I*_opt_ along the (b) *x* and (c) *y* axis for single domain (SD) and FSD samples with different thicknesses [104]. (d) Schematic of scattering scanning near‐field optical microscopy (s‐SNOM) and synchrotron infrared nanospectroscopy (SINS) measurements on a freestanding STO membrane. (e) Confinement factor of SPhPs in STO membranes and bulk as a function of frequency. (f) Propagation length (purple circles) and quality factor (blue squares) of SPhPs in a STO membrane versus frequency [105]. (g) Schematic of s‐SNOM/SINS on a suspended STO membrane. (h) SINS amplitude spectra s_2_(*ω*) from a line scan perpendicular to the membrane edge, showing SPhP fringes in Reststrahlen bands RB_2_ and RB_3_. (i) Lifetime (*τ*) of SPhPs in suspended STO versus thickness. Solid curves: calculations; symbols: experimentally extracted values [106]. Panels a‐c reproduced with permission: Copyright 2024, Wiley. Panels (g–i) reproduced with permission: Copyright 2024, Wiley.

Optical functionalities in correlated oxides often arise from the interplay between lattice symmetry, polarization, and electronic structure, all of which are reshaped in the freestanding limit. Correlated oxides host a rich array of optical phenomena, among which the bulk photovoltaic effect (BPVE) is a canonical case [103]. The BPVE (photocurrent generation in a homogeneous, non‐centrosymmetric crystal) operates without a built‐in junction field and thus contrasts sharply with conventional *p*–*n* photovoltaics. In representative BFO, ferroelectric order couples directly to the BPVE, providing robust levers for control. Removing substrate clamping in freestanding membranes markedly expands the tunability of polarization (magnitude, orientation, and domain topology), enabling stronger and more tunable BPVE responses. For example, single‐domain freestanding BFO membranes transferred onto Si substrates were adopted and showed an ≈200% enhancement of the bulk photovoltaic response relative to strained, substrate‐clamped films [104] (Figure 5a–c). The gain is attributed to enlarged Fe off‐centering, which strengthens the in‐plane ferroelectric polarization and thereby boosts the BPVE photocurrent.

Surface phonon polaritons (SPhPs) are interface‐bound excitations in polar dielectrics arising from photon–optical phonon coupling. On bulk crystals, they are weakly confined. Thinning to the deep‐subwavelength regime couples the two surfaces and splits SPhPs into symmetric and antisymmetric branches. Both branches confine fields far below the diffraction limit and enable infrared nanophotonics. Freestanding oxides also support intense, low‐loss phonon polaritons, and hold significant potential for waveguides, resonators, and metasurfaces. For example, Xu et al. showed that in 100 nm‐thick freestanding STO membranes placed on Si substrates, hybridization of the two surface phonon‐polaritons yields symmetric and antisymmetric branches, producing epsilon‐near‐zero (ENZ) and Berreman resonances and tenfold more tightly confined propagating phonon polaritons [105] (Figure 5d–f). This splitting stems directly from the membranes’ deep‐subwavelength thickness, demonstrating the large potential of transition‐metal oxide membranes as building blocks for future long‐wavelength nanophotonics. Recently, Koons et al. leveraged freestanding STO membranes on SiO_2_/Si windows to build suspended devices and, using near‐field techniques, systematically quantified far‐infrared SPhP propagation and loss [106] (Figure 5g–i). Decoupling from a lossy substrate yielded highly confined, low‐loss SPhPs with lifetimes up to ∼8 ps. In STO, SPhP energies span 30–50 meV, comparable to *k*_B_T (26 meV) at room temperature, making them readily thermally excitable and offering advantages over the higher‐energy mid‐infrared (IR) polaritons of conventional materials.

### 3.6 Magnetic Properties of Strain‐Free Membranes

Magnetism introduces an additional layer of complexity in freestanding oxides, because spin and orbital order are highly sensitive to subtle changes in lattice symmetry and bonding geometry. Magnetism in correlated oxides exhibits a rich spectrum of phenomena and device opportunities, including colossal magnetoresistance [107], multiferroicity [108], interfacial two‐dimensional electron gas (2DEG) [109] and magneto‐ionics [110, 111, 112]. In epitaxial films, substrate clamping, thermal mismatch, lattice distortions, threading defects, and oxygen‐vacancy gradients can all reshape the Curie temperature (*T*_c_), the saturation magnetization (*M*_s_), and the magnetic anisotropy by altering symmetry, strain, and interfacial electronic structure. Freestanding membranes eliminate this mechanical clamp, providing a route to probe intrinsic magnetism, to engineer it with greater freedom, and to integrate it into hybrid heterostructures.

**FIGURE 6:**
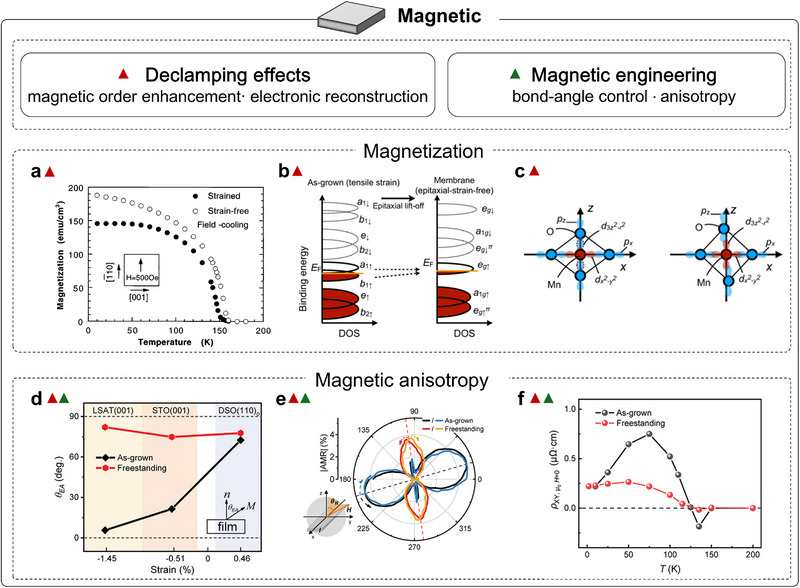
Magnetic properties of strain‐free oxide membranes. (a) Magnetization versus temperature for strained and strain‐relaxed SRO thin films [113]. (b) Schematic of tensile‐strain effects on the Mn 3*d* electronic structure in LSMO (solid line: majority spin; dotted line: minority spin). (c) Schematic of *p*–*d* hybridization in LSMO (red/blue circles: Mn/O); under tensile strain, bond lengths increase along *x* and decrease along *z* [114]. (d) Strain‐dependent magnetic easy‐axis angle, *θ*_EA_ for strained SRO films (black) and freestanding nanomembranes (red). (e) Angle‐dependent magnetoresistance of strained SRO on STO (001) (blue: clockwise; black: anticlockwise) and freestanding nanomembranes (orange: clockwise; red: anticlockwise). (f) Comparison of temperature‐dependent anomalous Hall resistivity (*ρ_xy_*) (e) for strained thin films on STO (001) and their corresponding freestanding nanomembranes [115]. Panel a reproduced with permission: Copyright 2024, AIP Publishing. Panels b and c reproduced with permission: Copyright 2024, American Physical Society. Panels d, e, and f reproduced with permission: Copyright 2022, Wiley.

This sensitivity was recognized early. In 1998, Gan et al. selectively etched away the substrate beneath SRO films and transferred onto LaAlO_3_ substrates to access the declamped state [113]. After strain relaxation, *T*_c_ increased by about 10 K (to ∼160 K), and *M*_s_ rose by about 20%, directly evidencing elastic‐strain control of SRO ferromagnetism. The enhancement was later attributed to relaxed strain modifying the Ru─O─Ru bond angles, bandwidth, and effective exchange [116] (Figure 6a), which drives the itinerant (near‐Stoner) ferromagnetism toward its bulk limit and elevates both *T*_c_ and *M*_s_. Follow‐up studies by varying crystallographic orientation, supporting platform, and residual strain further indicate that freestanding SRO placed on Si substrates approaches the low‐spin magnetic state characteristic of the bulk [117]. Except for SRO, the SAO_C_ water‐soluble sacrificial layer enables transfer of double‐exchange LSMO membranes onto polyimide tape in multiple crystallographic variants, which retain robust ferromagnetism after lift‐off [118]. The observed increase in *T*_c_ generalizes the “from declamping to enhanced magnetic order” conclusion from SRO to double‐exchange LSMO. Although SAO_C_ has enabled the fabrication of many freestanding oxides, its poor mechanical robustness often induces high crack densities in the released membranes. These cracks degrade functional properties, such as reducing the exchange‐bias strength in LSMO/BFO bilayers [119]. With the low‐symmetry tetragonal SAO_T_ phase, crack density in freestanding membranes is greatly suppressed, enabling micro‐scale studies of property evolution. For example, Takeda et al. used synchrotron measurements to probe why ferromagnetism is enhanced in a freestanding LSMO membrane after transferring onto Si substrates. They showed that strain release increases the itinerancy of Mn 3*d* electrons via strengthened *p*–*d* hybridization, which reinforces the double‐exchange interaction and thereby improves the ferromagnetism of LSMO [114] (Figure 6b,c). In addition, the stability and reliability of freestanding oxides are often limited by oxygen loss during transfer. Growing an oxygen‐rich capping layer of the same material under oxidizing conditions effectively suppresses vacancy formation and preserves stoichiometry, thereby stabilizing the functional properties of LSMO layer [120].

Magnetic anisotropy determines the easy axis, switching barrier, and resonance spectrum, directly governing energy cost and speed in spintronic, microwave, and magnonic devices. In freestanding oxides, lift‐off from substrate clamping turns anisotropy into a programmable route by reconstructing oxygen‐octahedral networks. In SRO, Peng et al. used a generic sacrificial‐layer route to obtain freestanding membranes on Si substrates and showed that anisotropy could be programmed by substrate‐imposed strain states and crystallographic orientations, with the observed easy‐axis changes from in‐plane to out‐of‐plane correlated to epitaxial‐strain‐induced octahedra rotations [115] (Figure 6d–f). In double‐exchange manganites, Arai et al. found that releasing LSMO from STO and placing it on Si substrates enhances the perpendicular component while weakening the in‐plane anisotropy, consistent with the suppression of substrate‐imposed strain and octahedral rotations [121]. Freestanding LaMnO_3_ (LMO) on PMMA further exhibits emergent uniaxial anisotropy in high‐integrity membranes, underscoring how strain relaxation and structural variants can reorient the easy axis [122]. Similarly, in freestanding BTO/LSMO bilayers supported on polyimide tape, the magnetic easy axis switches to out‐of‐plane, whereas the epitaxial counterparts remain in‐plane [123].

### 3.7 Superconducting Properties of Strain‐Free Membranes

Superconductivity offers a stringent probe of whether freestanding oxide membranes can preserve and tune collective correlated phases once substrate clamping is removed. In complex oxides, unconventional superconductivity is closely intertwined with strong electronic interactions and competing orders. Freestanding platforms therefore, provide an attractive setting for exploring superconductivity under programmable strain, cleaner interfacial conditions, and extreme dimensional control. An intriguing subset of cuprate superconductors, exemplified by Bi_2_Sr_2_CaCu_2_O_8__+_δ (Bi‐2212, often called BSCCO), is intrinsically layered and can be thinned by mechanical exfoliation while retaining high‐*T*_c_ superconductivity [124, 125]. Building on this platform, surface/ion transport has been used to reversibly tune carrier density and disorder, driving a controllable superconductor–insulator transition in exfoliated flakes [126]. By 2019, Yu et al. advanced extreme thinning with in situ encapsulation, pushing Bi‐2212 to the single‐layer (half‐uc) limit and demonstrating that superconductivity survives in the true 2D regime [46]. These results indicate that strong interlayer coupling is not required for superconductivity to emerge, and they establish a robust foundation for later vdW stacked homojunctions and heterojunctions based on cuprate membranes.

**FIGURE 7:**
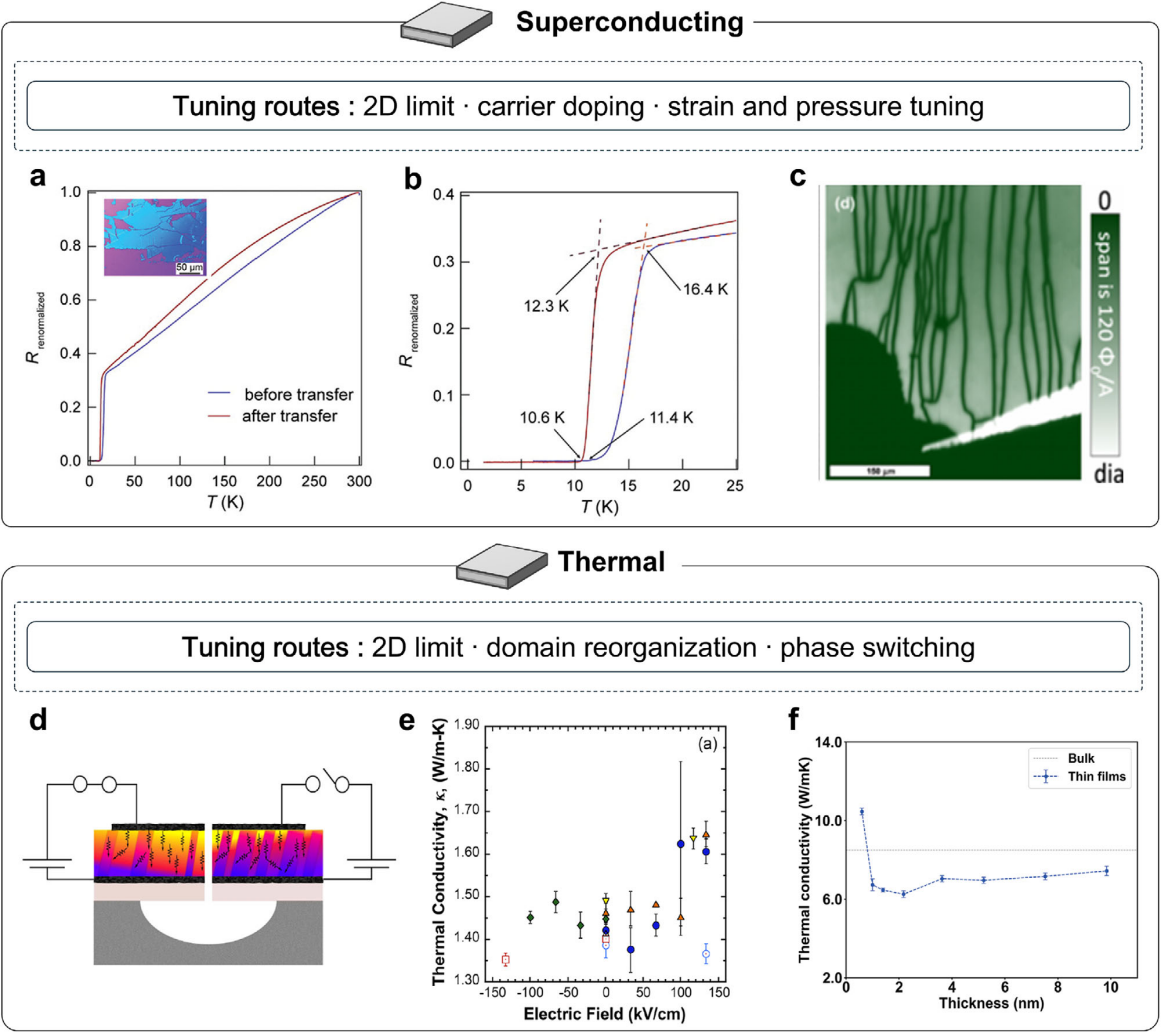
Superconducting and thermal properties of strain‐free oxide membranes. (a) Temperature dependence of resistance normalized to its 300 K value for the infinite‐layer film (before transfer) and the freestanding membrane (after transfer); inset, optical images of freestanding membranes on SiO_2_/Si. (b) Low‐temperature (0–25 K) normalized resistance traces [127]. (c) Scanning superconducting quantum interference device (SQUID) microscopy at 4.3 K showing a diamagnetic response (bright regions) [128]. (d) Schematic of a suspended ferroelectric PZT membrane. (e) Room‐temperature thermal conductivity of PZT for the suspended membrane device (closed symbols) and the clamped region (open symbols) versus applied DC field [42]. (f) Thermal conductivity of STO thin films of various thicknesses at 300 K [130]. Panels a and b reproduced with permission: Copyright 2024, Wiley. Panel c reproduced with permission: Copyright 2025, American Physical Society. Panels d and e reproduced with permission: Copyright 2018, American Chemical Society. Panel f reproduced with permission: Copyright 2023, American Physical Society.

For complex oxides grown by conventional epitaxy, releasing films from substrate clamping enables strain programmability, cleaner interfacial chemistry, and flexible dimensional control. It also allows extreme external stimuli that range from reversible bending to true hydrostatic pressure. These strategies enable a more direct remapping of the phase diagram, tuning pairing interactions, bandwidth, and carrier density in concert. For example, freestanding infinite‐layer nickelate membranes on Si substrates retain zero‐resistance transitions around 10–11 K after lift‐off and topotactic reduction, demonstrating that superconductivity can survive and be tuned in the declamped limit [127] (Figure 7a,b). Besides, electron‐doped cuprate single‐crystal membranes have also been fabricated in freestanding form and exhibit superconductivity, extending the applicability of the freestanding‐membrane platform across the high‐*T*_c_ oxide family [128] (Figure 7c). In parallel, high‐pressure transport platforms tailored for freestanding films eliminate substrate artifacts and permit clean tracking of phase evolution. For example, SrIrO_3_ (SIO) encapsulated by a ferroelectric nanoblock inside a diamond‐anvil cell withstands hydrostatic pressures up to ∼16.5 GPa without catastrophic cracking. With increasing pressure, semimetal‐to‐insulator and insulator‐to‐metal transitions are resolved, and in the monolayer limit, a direct insulator‐to‐metal crossover emerges [129]. These capabilities lay the groundwork for pressure tuning of future freestanding superconducting membranes, which may also enable pressure‐induced superconductivity.

### 3.8 Thermal Properties of Strain‐Free Membranes

Thermal transport in complex oxides is largely affected by lattice vibrations, while remaining highly sensitive to domain structures, defects, and structural phase transitions. In substrate‐clamped epitaxial oxides, thermal conductivity is strongly constrained by the imposed boundary conditions, with dense ferroelastic domain walls acting as internal phonon‐scattering centers. Releasing this clamping in freestanding membranes opens qualitatively new routes for phonon engineering, enabling larger modulation amplitudes and lower activation thresholds than are accessible in supported geometries. For example, switching the crystallographic phase of BFO from rhombohedral‐like (R‐like) to tetragonal‐like (T‐like) lowers thermal conductivity to about two‐thirds of the R‐like value at fixed thickness [131]. In epitaxial single‐crystalline BTO, time‐domain thermoreflectance combined with first‐principles modeling shows that *κ* decreases with thickness [132]. This shows that the heat is carried by phonons with mean free paths below ∼100 nm. Notably, with mean free paths longer than 10 nm still contribute ∼35% of the total heat flow. In contrast, protonated nickelates (H–ReNiO_3_) provide a different example [133]. Protonation drives a Mott transition that suppresses the electronic part of thermal conductivity. The result is a reversible, wide‐range reduction in thermal conductivity: LaNiO_3_ drops from ∼12.0 to ∼2.6 W m^−^^1^ K^−^^1^, and NdNiO_3_ from ∼8.0 to ∼1.6 W m^−^^1^ K^−^^1^. Overall, the substrate clamping in epitaxial systems imposes strong constraints, limiting the achievable modulation of thermal conductivity.

Declamping the epitaxial oxides as freestanding membranes opens qualitatively new routes of control. In suspended PZT placed over Si holes, ferroelastic walls reorganize abruptly at the coercive field, yielding a digital, bistable thermal conductivity jump of ∼13% at ∼100 kV cm^−^^1^, whereas supported PZT requires nearly 500 kV·cm^−^^1^ to reach a comparable ∼12.5% change with an analog response [42, 134] (Figure 7d,e). These enhancements arise from the release of substrate clamping, which promotes domain‐wall motion and coalescence. As the applied field approaches the coercive value, domains coarsen and spacing increases, reducing domain‐wall scattering and producing a sharp rise in thermal conductivity. Theory now extends this picture to freestanding STO down to the monolayer limit [130] (Figure 7f). It predicts that the thermal conductivity of the monolayer exceeds that of the bulk and a thickness trend where thermal conductivity first decreases from the monolayer, then approaches the bulk value as thickness increases, an evolution unattainable in the clamped geometry. Although fewer studies exist for membranes than for supported films, they consistently reveal larger contrast, lower thresholds, and new transport regimes beyond the substrate‐limited epitaxial baseline.

## 4 Exploration on Strained Complex Oxide Membranes

Freestanding oxide membranes are ultrathin structures, with low bending rigidity, reduced defect density, and accessible in‐plane/out‐of‐plane deformation. While micromechanical platforms enable precise probing of their flexural mechanics, directly applying controlled strain to suspended membranes is impractical for most experiments and devices. Instead, the standard route involves laminating the membranes onto flexible substrates (e.g., PDMS, PET), so that substrate deformation transfers strain across the interface. Flexible oxide devices serve as an essential complement to epitaxial, rigid‐substrate platforms. Two deformation regimes dominate, including uniform in‐plane tensile strain (uni‐ or biaxial) and out‐of‐plane bending that creates well‐defined strain gradients. Uniform strain reshapes bond lengths/angles and octahedral tilts, continuously tuning soft phonons, bandwidth, and symmetry, thereby modulating ferroelectric order, magnetic exchange, and charge transport. Bending introduces well‐defined strain gradients that couple to polarization through flexoelectricity to imprint local polarization and domain textures, enabling discrete or asymmetric control over electrical, magnetic, optical, and polaritonic responses. Together, these lattice‐engineering routes enable the tuning of polarization, symmetry, and phonon spectra, thereby reconfiguring thermal behavior. Declamping in membranes enhances coupling strengths and expands the accessible phase space beyond the epitaxial baseline.

### 4.1 Mechanical Properties of Strained Oxide Membranes

**FIGURE 8:**
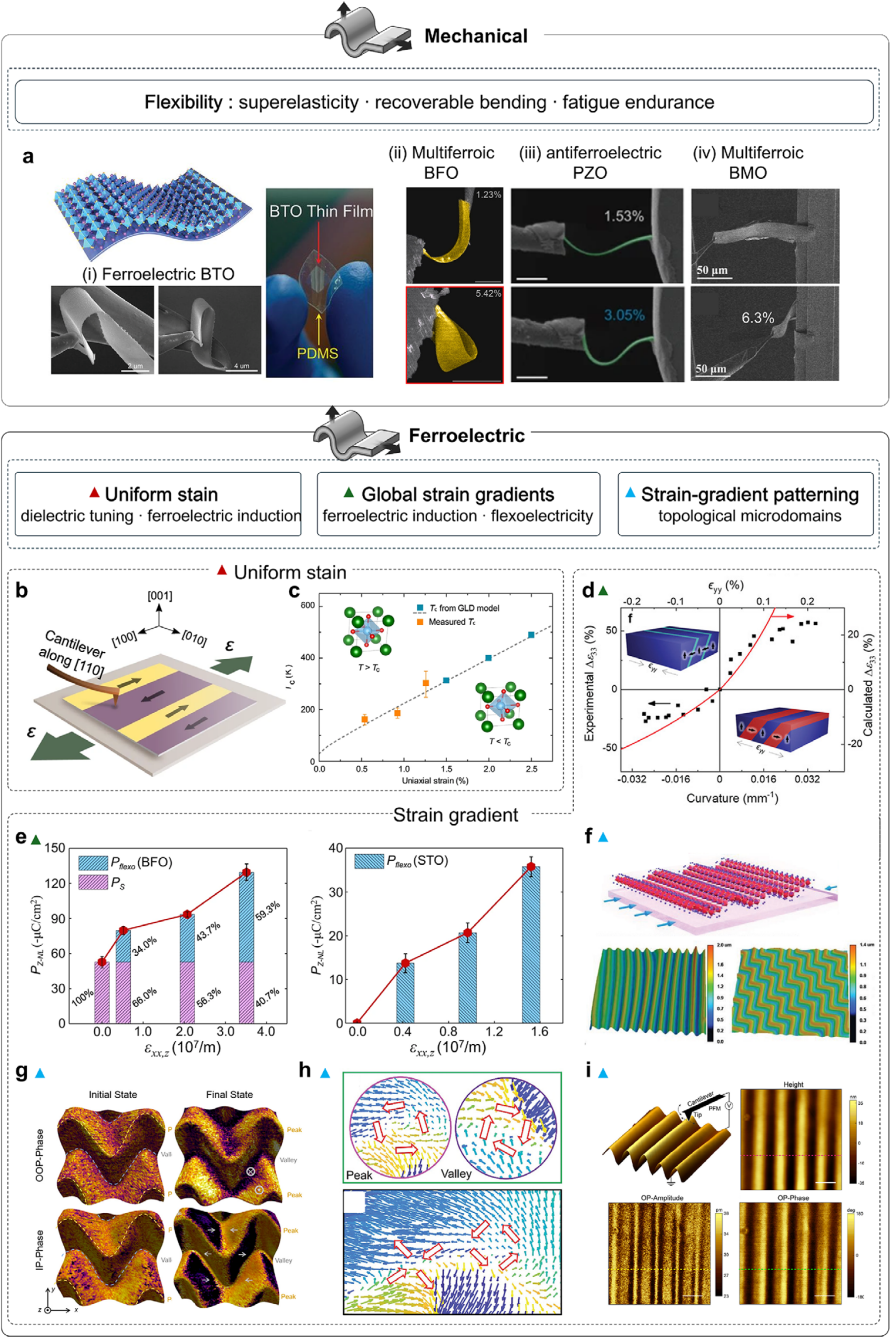
Flexible mechanical and ferroelectric properties of strained oxide membranes. (a) Schematic and optical photographs of a flexible BTO membrane (top and right). In situ SEM bending tests on freestanding nanobelts: (i) BTO [44], (ii) BFO [138], (iii) PZO [140], (iv) BMO [139]. (b) Schematic of PFM measurement on strained STO. (c) Ferroelectric transition temperature (*T*_c_) versus uniaxial strain from second‐harmonic generation (SHG) measurements, with high‐temperature extrapolations from the Ginzburg‐Landau‐Devonshire model [48]. (d) Measured (left axis) and calculated (right axis) bending‐induced dielectric tunability versus substrate curvature (bottom) and estimated bending strain (top); schematic drawings indicate expected domain configurations [142]. (e) Out‐of‐plane polarization at the neutral layer and corresponding strain gradients for bent BFO and STO membranes [144]. (f) Schematic (top) and laser‐scanning confocal images (bottom) of wrinkled BTO showing parallel and zigzag patterns [145]. (g) PFM phase‐color maps overlaid on 3D morphology and a schematic of tip‐induced domain switching [146]. (h) Vortex pairs and vortex–antivortex pairs observed in wrinkled BTO films [147]. (i) PFM responses of stripe‐wrinkled STO: 3D topography with out‐of‐plane PFM amplitude and phase [148]. Panels a‐i reproduced with permission: Copyright 2019, American Association for the Advancement of Science. Panels a‐ii reproduced with permission: Copyright 2020, American Association for the Advancement of Science. Panel d reproduced with permission: Copyright 1998, Wiley. Panel f reproduced with permission: Copyright 1998, Wiley. Panel g reproduced with permission: Copyright 2022, American Chemical Society.

Freestanding correlated oxide membranes enable genuinely substrate‐decoupled nanomechanics: once lifted off and suspended, they act as freestanding membranes or drum‐membranes in which deformation, elasticity, dissipation, and phase behavior can be read out free of clamping artifacts. The intrinsic deformability of a material defines how strongly its properties can be tuned by stress and thus how far a technology can ultimately scale. Bulk single‐crystal correlated oxides are notoriously brittle: under ambient conditions, they typically crack after only ∼0.1% elastic strain, which severely limits direct mechanical interrogation [135]. By contrast, coherently grown epitaxial films can sustain percent‐level biaxial strains (≈1%–3% is routine below the critical thickness) [135]. The advent of freestanding oxide membranes, which enable direct nanomechanical testing, now allows intrinsic deformation mechanics to be probed. In the uniform‐strain regime, Hong et al. used freestanding La_0.67_Ca_0.33_MnO_3_ (LCMO) to reach extreme elongations of 5% biaxial and ∼8% uniaxial, providing a broad window to tune coupled electronic and magnetic properties [49]. By contrast, bending imposes controlled strain gradients, unlocking richer structural distortions and additional pathways for functionality. In 2019, Dong et al. performed in situ bending/folding tests on freestanding BTO single‐crystal membranes [44] (Figure 8a), showing nearly 180° folding without fracture and recoverable bending strains approaching ∼10%. The superelasticity is attributed to continuous dipole rotation and domain reconfiguration rather than conventional 90° domain switching during bending. Phase‐field simulations further show how bending angle and thickness drive transitions from flux‐closure patterns to a/c domains with vortex‐like structures [136]. Beyond planar stretching and bending, strain‐driven self‐assembly can further convert freestanding epitaxial heterostructures into 3D spring geometries that dramatically expand the reversible deformation window. Dong et al. reported self‐assembled, epitaxial LSMO/BTO ferroelectric oxide nanosprings formed from freestanding bilayers with built‐in mismatch strain, which can be repeatedly pulled or pushed to their geometric limits with full recovery and a reported scalability up to ∼500% [137]. Soon after, Peng et al., extended superelasticity from BTO to a multiferroic oxide BFO. They identified a reversible phase transition between rhombohedral and tetragonal structure as the key strain‐accommodation channel in BFO, enabling cyclic 180° folding and maximum bending strain of ∼5.42% without cracking [138]. This superflexible property is further verified in more and more oxides, including multiferroic BaMnO_3_ (BMO) (∼6%) [139] and antiferroelectric PbZrO_3_ (PZO) (∼3%) [140] (Figure 8a). Interestingly, the strain‐release mechanism of antiferroelectric PZO includes a reversible transition between antiferroelectric (AFE) and ferroelectric (FE) phases together with the polarization rotation, filling a missing corner of the ferroic map. Beyond static strength, nanoindentation of antiferromagnetic membranes quantified remarkable robustness, with elastic strains approaching ∼6% and endurance over ∼10^9^ fatigue cycles at ∼85% of the fracture strain [141], underscoring suitability for durable nanoelectromechanical systems (NEMS).

### 4.2 Ferroelectric Properties of Strained Oxide Membranes

Ferroelectricity is highly sensitive to structure, making mechanical actuation an efficient and convenient control modality. Integrating freestanding ferroelectric oxides with flexible substrates, therefore offers a powerful platform for mechanically driven polarization control. Based on uniform in‐plane deformation, Xu et al. successfully induced ferroelectricity in STO by applying 2.0% uniaxial tensile strain, verified by optical SHG [48] (Figure 8b,c), driving quantum‐paraelectric STO into room‐temperature ferroelectricity in 2020. The appearance of 180° domains and an extrapolated transition temperature near 400 K further corroborate the strain‐driven polar phase. In the same year, in situ stretching on a polymer substrate revealed pronounced, strain‐induced permittivity modulation, showing ∼90% tunability at room temperature under only ±0.1% uniform in‐plane strain [142] (Figure 8d). Interestingly, on this platform a low‐temperature classical‐to‐quantum crossover of the ferroelectric transition was observed under uniaxial tensile strain (>1% with reversible cycling), revealing a strain‐tunable entry into the quantum regime [143]. Besides, Huang et al. used SHG to map ferroelectric order in freestanding PbTiO_3_ (PTO) membranes under continuously varied tensile strain and temperature [144] (Figure 8e). Unlike epitaxial PTO, which exhibits only out‐of‐plane domains, the freestanding films host both out‐of‐plane and in‐plane polar textures. Tensile strain strengthens polarization from 0% to 1.66%, and even under large biaxial tension (*ε*_x_ ≈ 2.76%, *ε*ᵧ ≈ 4.46%) the membranes retain an orthorhombic‐like phase with robust ferroelectricity, highlighting their promise for flexible devices.

Strain gradients introduced by bending can directly lower crystal symmetry, offering a more effective and convenient handle on ferroelectricity than uniform strain. Using atomically resolved STEM on few‐nanometer freestanding BFO and STO membranes, Cai et al. quantified curvature‐induced gradient and polar displacements, showing that nanoscale bending generates ∼10^7^ m^−^^1^ strain gradient and markedly enhances polarization [85]. In BFO, local surface strain reaches ∼7.8%, yielding a maximum gradient of ∼3.5 × 10^7^ m^−^^1^. These results highlight a distinctive advantage of freestanding membranes: a large, thickness‐uniform strain gradient across the film during bending. Leveraging declamping and orientation engineering in freestanding PZT membranes markedly boosts dielectric and piezoelectric performance [100]. Flexible PENGs built from these films deliver a volumetric power density of ∼63.5 mW cm^−3^, an effective *d*_33_ ≈ 585 pm·V^−1^, a strain tolerance >3.4%, and > 60000‐cycle stability (open‐circuit voltage up to 12 V), underscoring strong application potential.

Beyond global bending, the low out‐of‐plane stiffness of ultrathin films makes them prone to buckling under in‐plane compression, producing ripple/wrinkle topographies that localize curvature. On freestanding single‐crystal BTO laminated to PDMS, Dong et al. used controlled compression to generate three ordered wrinkle arrays, parallel, zigzag, and mosaic [145] (Figure 8f). The wrinkle geometry concentrates curvature and stress at crests and troughs, creating a spatially periodic strain‐gradient field. Those gradients induce a local flexoelectric polarization proportional to the gradient and, in a ferroelectric, add vectorially to the switchable spontaneous polarization. The crest–trough pattern thus becomes an addressable set of polar/piezoelectric “hot spots,” turning ordered wrinkles into a programmable template for strain‐gradient engineering. Building on this platform, Zhou et al. used the local stress/strain gradient of an AFM tip to superimpose and steer domains atop the pre‐existing wrinkle field, yielding reproducible, woven in‐plane polarization superstructures, marking a transition from purely geometry‐set patterns to actively written local architectures [146] (Figure 8g). Later, Wang et al. used a sawtooth wrinkle array to stabilize vortex–antivortex pairs and head‐to‐head/tail‐to‐tail boundaries under an ultralow applied strain (≈0.5%), with 3D vector piezoresponse force microscopy (PFM) and phase‐field simulations, showing one‐to‐one correspondence [147] (Figure 8h).

Intriguingly, pre‐stretch–release processing of non‐polar STO nanomembranes creates stripe wrinkles whose strain gradients generate flexoelectric polarization, enabling the creation and control of ordered polar stripes in an otherwise non‐ferroelectric crystal. In situ PFM shows that increasing the applied tensile strain amplifies wrinkle amplitude and wavelength, thereby tuning the gradient and the polarization strength [148] (Figure 8i). Complementary theory traces an evolutionary pathway, from non‐polar to polar stripes, then to meron/antimeron textures and ultimately labyrinthine‐like domains, yielding a geometry‐flexoelectric phase‐diagram perspective that underpins wrinkle‐engineered ferroelectric design in freestanding oxides [149]. Recently, freestanding PTO membranes that spontaneously form periodic ripples were shown to use curvature to locally select polarization and domain texture [150]. Crests host purely in‐plane superdomains, whereas troughs and flats display mixed in‐plane/out‐of‐plane patterns. This spatial partitioning demonstrates that geometric curvature can deterministically select and stabilize distinct polar/domain states within ordered ripple arrays. Interestingly, recent work shows that local dome‐like deformations can generate strain‐gradient fields that induce topological polarization [52]. In freestanding BTO membranes a few to tens of nanometers thick, micrometer‐scale domes drive the polarization vectors to converge radially toward the dome center, forming stable center‐convergent topological microdomains. These microdomains act as nonlinear and electro‐optic phase plates, enabling spatial beam shaping, including orbital angular momentum, and vortex conversion/modulation of second‐harmonic generation. The underlying mechanism is anisotropic lattice distortion within the dome that produces a radial strain gradient; via flexoelectric coupling. This gradient then induces an inward effective field that stabilizes the center‐convergent domain pattern, effectively “winding” the polarization into the core through strain and curvature. These examples show that mechanical programming of polar textures is inseparable from metrology and boundary‐condition control. Across these platforms, quantitative comparisons require calibrated mapping of strain and curvature, together with controlled electrostatic boundary conditions that govern screening and domain stability. Demonstrating fatigue‐resistant operation under repeated bending and electrical cycling will be critical for translating gradient‐programmed polar states into reliable flexible devices.

### 4.3 Magnetic Properties of Strained Oxide Membranes

**FIGURE 9:**
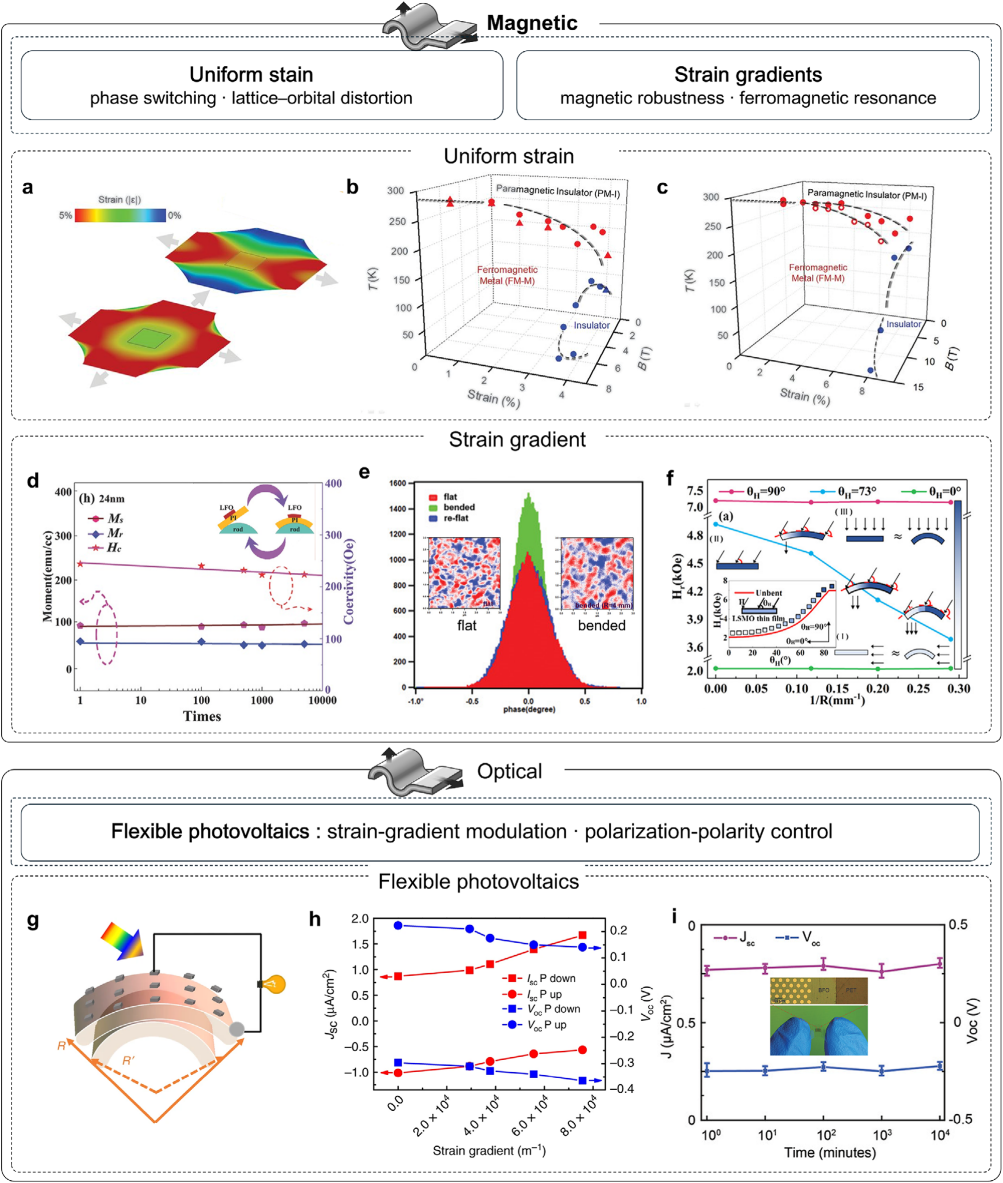
Flexible magnetic and optical properties of strained oxide membranes. (a) Finite‐element strain profiles of the polyimide/oxide bilayer under uniaxial and biaxial uniform stress. (b) Phase diagram of a biaxially strained LCMO membrane. (c) Phase diagram of a uniaxially strained LCMO membrane [49]. (d) Plots of *M*_s_, *M*_r_, and *H*_c_ versus bending cycle count for a 24 nm transferred film [151]; inset, schematic of the bending‐cycle test. (e) Histogram of the magnetic force microscopy (MFM) phase for an Fe_3_O_4_ thin film in flat, bent, and re‐flattened states [152]; inset, MFM phase maps in flat and bent. (f) Resonance field for a 50 nm unbent LSMO thin film under various bending states [153]; inset, angular *θ*_H_ dependence of *H*_r_ for the unbent film. (g) Schematic of the realization of the strain gradient by bending the freestanding BFO thin film. (h) The corresponding change of *V*_oc_ and *J*_sc_ as a function of the introduced in‐plane strain gradient for both polarizations [154]. (i) *V*_oc_ and *I*_sc_ of freestanding single‐crystalline BFO membrane‐based photovoltaic devices versus rest time. Inset, optical image of the single‐crystalline BFO membranes‐based photovoltaic devices [155]. Panels a, b, and c reproduced with permission: Copyright 2020, The American Association for the Advancement of Science. Panel d reproduced with permission: Copyright 1998, Wiley. Panel e reproduced with permission: Copyright 2001, Wiley. Panel f reproduced with permission: Copyright 2019, Wiley.

Releasing the films and exploiting strain tunability demonstrates that magnetism is highly adjustable. Pairing freestanding oxides with flexible substrates enables truly flexible magnetic devices. Two modalities dominate: uniform (biaxial/uniaxial) strain and bending‐induced uniaxial strain, enabling phase control, anisotropy reorientation, and ferromagnetic resonance (FMR) tuning for flexible spintronics, magnetoelectrics, and microwave components. Under uniform strain. Hong et al. used freestanding LCMO to access extreme uniform strains, up to >5% biaxial and >8% uniaxial, and discovered a switch from ferromagnetic metallic to insulating‐antiferromagnetic state above a critical biaxial strain (3%) [49] (Figure 9a–c). The transition is reversible under the magnetic field and is attributed to strain‐driven Jahn–Teller distortions that reduce orbital overlap. This establishes that elastic strain alone can toggle correlated ground states in oxide membranes, defining the operating space for mechanical modulation.

Flexible devices, with low out‐of‐plane stiffness, unavoidably experience bending strain. Like other freestanding oxides, magnetic membranes withstand large curvature without fracture and often retain, or even enhance their magnetic performance. Large‐area ferrite membranes illustrate the point: centimeter‐scale single‐crystal LiFe_5_O_8_ films, lifted off and transferred to polyimide, maintain magnetism under bending and cycling, providing fatigue‐resistant, device‐ready formats [151] (Figure 9d). Freestanding Fe_3_O_4_ membranes push compliance further, bending radii down to ∼7 µm and twists ∼122°, while preserving robust magnetic response [152] (Figure 9e). Besides, bilayer LSMO/BFO stacks transferred onto PDMS retain a strong exchange bias on the LSMO layer [119]. Their saturation magnetization and exchange‐bias field remain essentially unchanged after 1000 bending cycles with a bending radius of ∼2 mm, underscoring mechanical robustness and device viability. In addition, freestanding BTO/(Co/Pt) stacks display excellent flexibility and malleability, with an FMR field shift of ∼440 Oe at a 5 mm bending radius [156]. Besides, freestanding LSMO: NiO composites transferred to polymer maintain perpendicular interfacial exchange bias with no degradation after extensive bending, adding vertical‐interface control of anisotropy to the freestanding toolset [157]. Finally, superflexible BMO membranes keep ferroelectricity and ferromagnetism under large curvatures, pointing to mechanically reconfigurable multiferroics [139].

On these flexible platforms, microwave magnetism becomes tunable by curvature. In 50‐nm LSMO, the FMR remains invariant near the magnetic field angle of 0°, 90°. yet shows strong, continuous shifts at intermediate angles when bent, demonstrating angle‐dependent microwave tunability in a single crystalline film [153] (Figure 9f). Ni_0_._5_Zn_0_._5_Fe_2_O_4_ (NZFO) membranes achieve strain‐tunable FMR shifts exceeding ∼2000 Oe, highlighting the potential for reconfigurable RF/microwave devices [158]. Interestingly, freestanding antiferromagnetic α‐Fe_2_O_3_ membranes are highly flexible, sustaining curvatures up to ∼3 × 10^−4^ nm^−1^ and even 180° folding. This enables 3D, programmable strain fields that reconfigure the antiferromagnetic state [159]. Overall, uniform tensile/compressive fields set the phase/ground‐state landscape (up to a few percent strain), while bending offers local, reversible anisotropy and FMR control on device‐ready flexible substrates. Together they deliver low‐energy, on‐chip strain programming of magnetic properties that are hard to achieve in clamped epitaxy.

### 4.4 Optical Properties of Strained Oxide Membranes

Declamping strengthens the intrinsic polarization, and strain relaxation yields a pronounced enhancement of the BPVE. This highlights the powerful strain tunability of BPVE in flexible device architectures. Guo et al. investigated transferred Pt/BFO/LSMO devices and found that reversing BFO polarization flips the photovoltage polarity, evidencing a ferroelectric photovoltaic response driven by the polarization‐induced internal field [154] (Figure 9g,h). Bending the freestanding membrane imposes a tunable strain gradient that generates a flexoelectric field; superposed with the intrinsic field that underlies the BPVE, it continuously modulates carrier‐separation efficiency, photoconductance, and photovoltaic output, yielding multilevel photoconductive states reproducible after ≥ 300 bending cycles. This demonstrates the power of mechanical control in flexible ferroelectric optoelectronics. Furthermore, Qiu et al. fabricated freestanding SRO/BFO membrane stacks, using SRO as the bottom electrode for reliable electrical access and integrated them onto a flexible, FET‐compatible substrate to realize bendable BPVE devices with stable sensing performance [155] (Figure 9i).

## 5 Development in Heterostructures Based on Freestanding Oxides

### 5.1 Heterogeneous Integration of Oxide Membranes

vdW interfaces formed by stacking layered 2D crystals, such as graphene, hBN, and TMDs, offer capabilities beyond conventional epitaxy. Because layers couple via vdW forces rather than covalent bonds, there are no dangling bonds, misfit dislocations or alloyed interphases, enabling atomically sharp, chemically inert contacts and “Lego‐like” lattice integration [160, 161]. Dielectric hBN encapsulation suppresses charged‐impurity and remote‐phonon scattering, restoring near‐intrinsic transport [162]. The dielectric environment also resets Coulomb screening and exciton binding energies, allowing optical spectra and many‐body interactions to be tuned without chemical modification [163, 164]. At 2D/metal contacts, weak metal‐induced gap states reduce Fermi‐level pinning, so Schottky barriers and contact resistance are tunable by electrode choice, gating, and stacking order [165, 166, 167]. The weak directionality of vdW bonding also introduces a twist degree of freedom: controlled angles create moiré superlattices that reshape bands, yielding flat‐band physics, correlated insulators, unconventional superconductivity, and interlayer excitons [168, 169]. Finally, interlayer spacing could serve as a practical control parameter, while the intrinsically low, tunable thermal boundary conductance can be exploited for heat management and phonon engineering [170].

Once lifted off and transferred, freestanding oxide membranes can be integrated with other oxides or layered 2D crystals to form atomically sharp, low‐damage vdW interfaces. Oxide‐based vdW interfaces share the generic benefits of layered 2D/2D stacking. Adjacent crystals meet without covalent bonding. Misfit dislocations and alloyed interfacial phases are therefore suppressed. Dissimilar lattices can also be assembled in a modular manner into atomically sharp, chemically inert contacts. Furthermore, oxide‐involved junctions retain key advantages while introducing interface phenomena rooted in coupled electronic, spin, lattice, and orbital degrees of freedom, along with corresponding challenges, mainly including:

- Dielectric oxides: High‐*κ* electrostatics: When freestanding high‐*κ* perovskite oxides serve as gate dielectrics in 2D MOS transistors, the ångström‐scale atomically clean quasi‐vdW gap acts as a tunneling barrier that strongly suppresses leakage. For example, despite the moderate bandgap of STO (indirect bandgap ∼3.3 eV), the vdW gap enables leakage currents below 10^−^^6^ A·cm^−^^2^, reducing static power consumption [171]. Simulations further show that this interfacial gap mitigates fringing‐induced barrier lowering (FIBL), preserving electrostatic control in scaled channels. The interface trap density at the STO/MoS_2_ interface remains on the order of 10^1^ ^2^ cm^−^^2^·eV^−^^1^, comparable to conventional high‐κ dielectrics but higher than pristine vdW insulators (∼10^1^ ^0^ cm^−^^2^·eV^−^^1^) [171]. These traps originate mainly from oxygen‐vacancy–related donor states, which capture and release channel electrons, producing transfer‐curve hysteresis and limiting stability. Further advances in defect passivation and transfer cleanliness are essential to unlock the intrinsic dielectric potential of freestanding perovskite membranes for low‐power, high‐performance 2D electronics.
- Ferroelectric oxides: polarization effect: In ferroelectric–2D heterostructures, bound surface polarization charges couple across an ångström‐scale vdW gap to the low‐density of state (DOS) channel. As a result, the depolarization field, memory window, and retention are set by the series combination of the channel's quantum capacitance and the ferroelectric dielectric capacitance. A clean vdW interface that minimizes residue, intermixing, water, and ionic adsorbates suppresses interface traps. Band alignment is then set mainly by work functions and built‐in dipoles, which yield tight device statistics and stable thresholds. A geometrically well‐defined sub‐nanometer gap restores exponential tunneling while minimizing redox‐driven drift and vacancy migration. These principles underpin non‐volatile, multistate operation and deterministic polarization control in ferroelectric‐gated 2D transistors.
- Magnetic oxides: exchange and spin‐orbit proximity: Releasing magnetic oxides from epitaxial clamping into transferable membranes transforms the interface into an active arena where strain‐, exchange‐, and twist‐mediated couplings interplay. Declamping allows nearly complete transfer of piezoelectric strain into magnetostrictive layers such as LSMO, strengthening magnetoelastic coupling and reducing the fields required for magnetization reversal. Clean, weakly bonded vdW contacts preserve low‐defect electronic structures while supporting exchange proximity, as evidenced by valley polarization imprinted by freestanding LMO on monolayer WS_2_. Magnetic anisotropy becomes programmable through strain transfer or interlayer twist, reshaping the in‐plane strain tensor and magnetocrystalline energy. Beyond oxide–oxide stacks, exchange and spin‐orbit proximity effect in oxide/2D vdW heterostructures depend sensitively on cleanliness and registry, with twist‐angle‐dependent SOP providing quantitative design targets for membrane‐assembled spin logic and spin‐photonic devices.
- Thermal oxides: SPhP and phonon‐tunneling: At vdW oxide/2D interfaces, weak interlayer bonding yields a small force constant and low phonon transmission, giving thermal boundary conductance (TBC) values typically below those of covalent contacts. Combined with the high in‐plane thermal conductivity of 2D layers or freestanding oxides, this produces strong thermal anisotropy. As the gap narrows to the nanometer–ångström scale, near‐field channels emerge: SPhPs and evanescent phonon tunneling transmit heat across the gap, particularly in polar oxides such as STO. In metal/ferroelectric stacks, interfacial charge and field‐dependent phonon spectra enable electrical or strain tuning of TBC in select systems. Clean, well‐defined interfaces make the thermal pathway programmable, yet contamination or roughness rapidly degrades coupling. Thus, achieving atomically sharp and stable vdW gaps is essential for balancing isolation and efficient cross‐plane heat dissipation in oxide/2D heterostructures.

### 5.2 Multifunctional Heterostructures

#### 5.2.1 Dielectric Oxide Heterostructures: High‐*κ* Oxide/TMD

**FIGURE 10:**
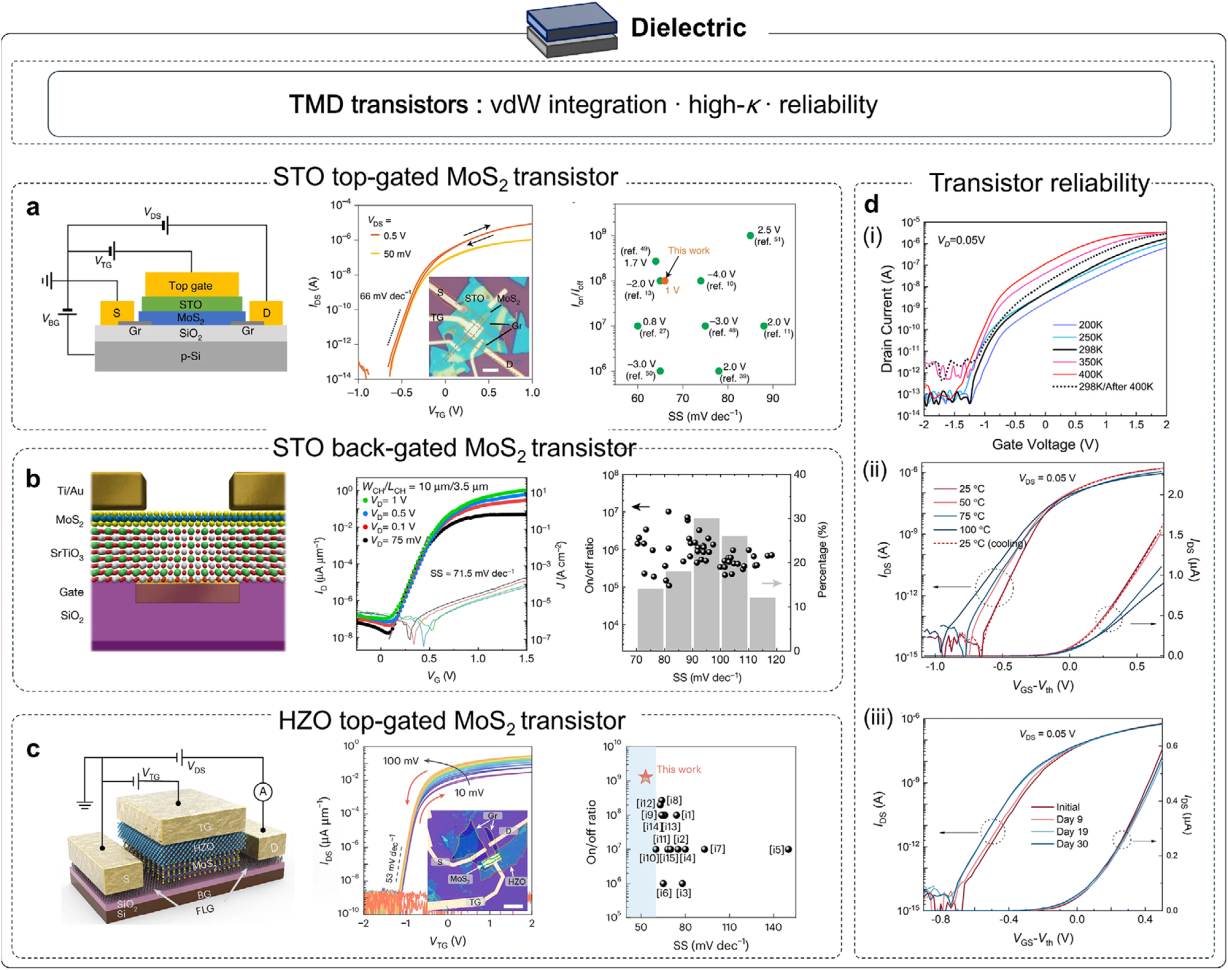
High‐κ transistors enabled by freestanding oxides. (a–c), From left to right: device schematics, representative *I*_DS_–*V*_G_ characteristics and on/off ratios of MoS_2_ FETs using STO as (a) a top‐gate dielectric [40] and (b) a back‐gate dielectric [41], and using (c) HZO as a top‐gate dielectric [175]. (d) Temperature dependence of the *I*_D_–*V*_G_ characteristics for MoS_2_ FETs using STO as a (i) back‐gate dielectric [92] and (ii) a top‐gate dielectric. (iii) Evolution of transfer characteristics with storage time in ambient air at room temperature for top gate STO FET [174]. Panels b reproduced with permission: Copyright 2022, Springer. Panels d reproduced with permission: Copyright 2025, Royal Society of Chemistry (Great Britain).

In microelectronics, oxides derive much of their value from their role as high‐*κ* gate dielectrics, from hafnia (HfO_2_, *κ* ≈ 20–25) to strontium titanate (STO, *κ* ≈ 300 at 300 K), which enable large gate capacitance and low‐voltage operation and are widely viewed as key candidates for the post‐Moore era. In 2022, Yang et al. and Huang et al. independently realized MoS_2_ n‐FETs using STO as top‐gate and bottom‐gate dielectrics [40, 41], respectively, achieving ultrahigh on/off ratios of ∼10^8^ and ∼10^7^ and ultralow subthreshold swings of 66 and 70 mV·dec^−^^1^ (Figure 10a,b). Complementary WSe_2_ p‐FETs with STO dielectrics likewise reached on/off ratios >10^7^, underscoring STO's promise for p‐type channels. Building on these devices, low‐power complementary MOS (CMOS) inverters were demonstrated with static power consumption at the picowatt level. Additionally, the vdW gap at the STO/2D‐channel interface suppresses electron tunneling [172, 173], thereby mitigating field‐induced barrier lowering in short‐channel, ultrahigh‐*κ* devices. The 35 nm MoS_2_ field effect transistors with STO dielectrics show clean optical morphology and characteristic transfer curves, with on/off ratios >10^5^ and subthreshold swings as low as 79 mV·dec^−^^1^. A comparison across short‐channel FETs highlights that STO‐based, vdW–integrated MOS transistors offer latent advantages over conventional architectures. Moreover, STO‐gated FETs exhibit notable stability and reliability. Sattari‐Esfahlan et al. [92] and Liu et al. [174] evaluated bottom‐ and top‐gated MoS_2_ devices, respectively, and found virtually no performance degradation across 76–400 K or after eight months of ambient storage, attesting to STO's robustness as a gate dielectric (Figure 10d).

Except for STO, industry‐aligned HZO has likewise been integrated as a high‐*κ* dielectric [175]: 20 nm HZO membranes exhibit a dielectric constant of 20.6 ± 0.5 and a leakage current below 2.6 × 10^−^^6^ A·cm^−^^2^ at 1 MV cm^−^^1^, while MoS_2_ transistors gated by HZO achieve on/off ratio of 10^9^ and subthreshold swings below 60 mV dec^−^^1^ across four decades of current (Figure 10c). Taken together, these advances establish vdW integration as a versatile route to high‐*κ* transistor platforms to deliver steep switching, large on/off ratios, low leakage and robust reliability, while maintaining scalability and compatibility with flexible and Si‐based technologies, underscoring the broader promise of oxide vdW integration for next‐generation electronics.

#### 5.2.2 Ferroelectric Oxide Heterostructures: Oxide/Si, Oxide/TMD, and Oxide/Oxide

**FIGURE 11:**
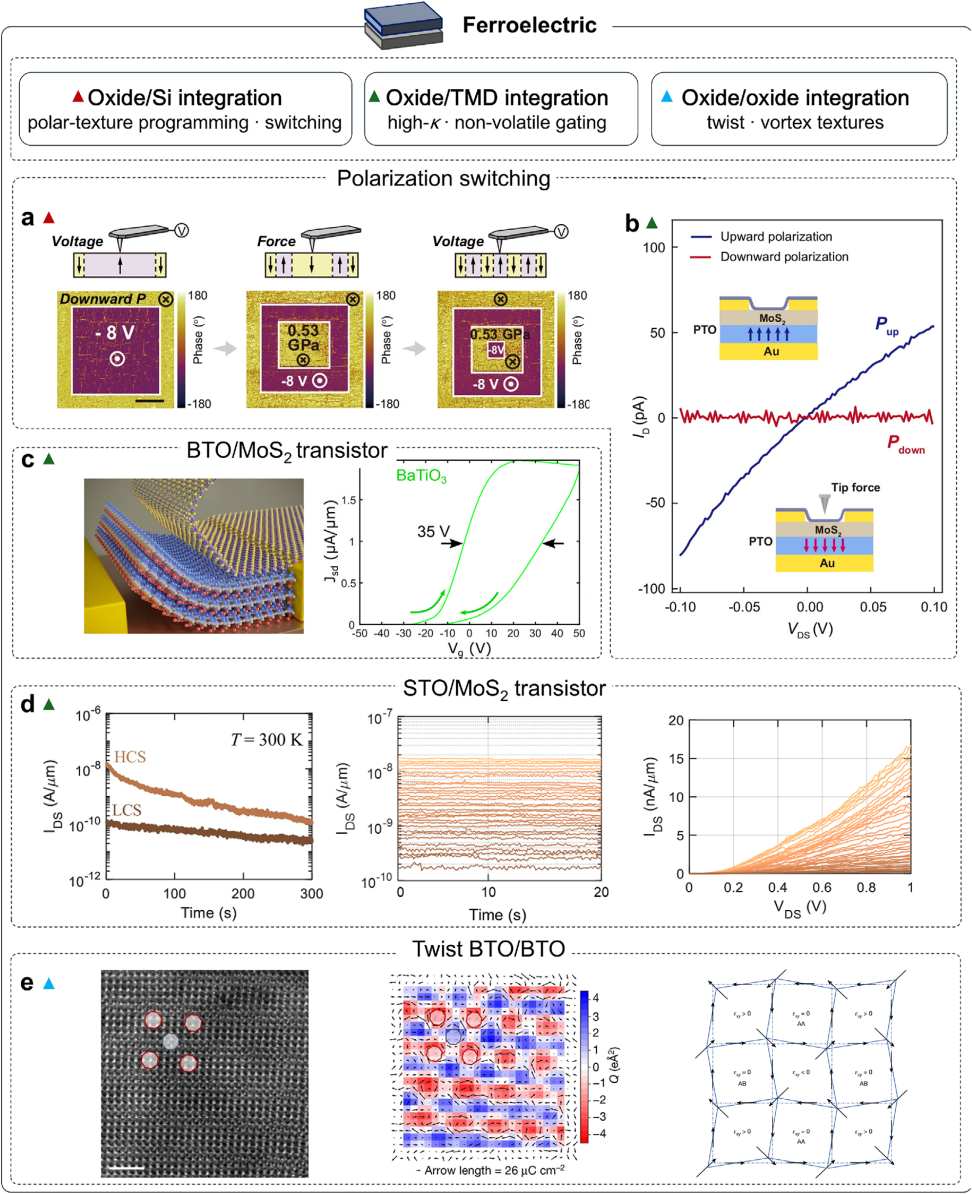
Ferroelectric properties of oxide heterostructures. (a) Vertical‐PFM phase images of PTO/Au/Si after electrical writing (left), mechanical erasing (middle), and electrical rewriting (right). Scale bar, 2 µm. (b) Output characteristics of a device with the PTO membrane preset to ***P*_up_** or ***P*_down_** (*V*_GS_ = 0 V) [177] (c) Schematic of a BTO/MoS_2_ transistor and representative transfer hysteresis loops [178]. (d) Incipient ferroelectricity STO‐gated MoS_2_ FET. From left to right: Non‐volatile retention of memory states at 300 K, thirty‐two memory states realized by programming pulses of fixed width (10 ms) and varying amplitude (5‐bit storage) and corresponding output characteristics [179]. (e) Moiré‐enabled ferroelectric vortex formation in twisted freestanding BTO. From left to right: Plan‐view HAADF‐STEM image showing a moiré superlattice, reconstructed in‐plane polar‐displacement field with the toroidal‐moment map revealing a vortex–antivortex lattice, and a schematic showing twist‐induced modulated shear strain and strain gradients that couple to polarization via flexoelectricity [45]. Scale bar, 2 nm.

Si integration offers the most direct route to scalable microelectronic deployment because it provides mature wafer‐scale manufacturing, dense on‐chip routing and readout, and established reliability qualification. It also supplies a well‐developed device and metrology infrastructure for benchmarking and system‐level integration beyond standalone material demonstrations. Ferroelectric oxides add a distinct capability on Si because switchable polarization imposes an internal electrostatic boundary condition that reconfigures band bending and carrier distributions at ferroelectric/semiconductor interfaces. This enables non‐volatile, field‐programmable junction behavior and texture‐enabled functionalities that passive dielectrics do not provide. In 2022, Sun et al. transferred BTO onto doped Si and showed that depolarizing fields and strain relaxation rotate the as‐grown, purely c‐axis polarization into in‐plane multidomain states [176]. Harnessing this robust in‐plane order, conductive domain walls with nanoampere‐level read currents were deterministically written and erased, highlighting the promise of perovskite/Si domain‐wall memories. Besides, Han et al. fabricated PTO/STO bilayers on Si, where room‐temperature skyrmion‐like polar nanodomains emerge [50]. Applying an external electric field switches these nanodomains between distinct polar textures, resulting in pronounced, reversible resistance modulation. The resistive response is attributed to the different band bending and charge carrier distributions associated with each polar configuration. Recently, Yang et al. reported a freestanding ferroelectric PbTiO_3_ membrane with ultralow pressure (∼0.06 GPa) mechanical polarization switching on Si platform [177] (Figure 11a,b). Collectively, ferroelectric oxide/Si integration elevates freestanding membranes from isolated model systems to platform‐ready building blocks, linking programmable polarization textures to Si‐compatible device concepts with clear pathways toward scalable manufacturing.

Ferroelectric oxides can be integrated with TMDs in the same transistor geometry used for dielectric oxides, enabling ferroelectric‐gated FETs. Compared with oxide/2D dielectric stacks, ferroelectric oxide/2D stacks add switchable polarization. This enables non‐volatile threshold control and yields distinct hysteresis and switching behavior. A representative demonstration of this architecture was reported by Puebla et al., who built a transistor in which a freestanding ferroelectric perovskite directly gates a 2D semiconductor [178] (Figure 11c). Single‐crystal BTO membranes were lifted off and transferred onto monolayer MoS_2_ to realize a ferroelectric FET, where BTO polarization enables non‐volatile threshold control and improves both the subthreshold swing and on/off ratio. Subsequently, Wu et al. systematically investigated freestanding PZT membranes with different top/bottom electrodes (metallic Au), the correlated oxide LSMO, and the 2D semiconductor MoS_2_, showing how material parameters govern ferroelectric response and domain stability and elucidating the underlying mechanisms [180]. Interestingly, integrating PTO with MoS_2_ to form a transistor yields a mechano‐electric, dual‐mode, non‐volatile ferroelectric field effect transistor (FeFET). The freestanding membrane's bendability and controllable strain gradients introduce a strain‐induced‐polarization (flexoelectric) degree of freedom into hybrid 3D/2D FeFETs, enabling ultra‐low‐energy operation and unified stress‐sensing–memory architectures. Beyond canonical ferroelectrics, the incipient ferroelectricity of STO has been employed as a gate dielectric in MoS_2_ transistors (Figure 11d). Between 100 and 15 K, STO develops stable ferroelectric order, enabling non‐volatile memory; at room temperature, its weak, marginally switchable polarization provides the fading memory needed for hardware reservoir computing. The integration scheme maintains a ∼180°C total thermal budget, compatible with back‐end‐of‐line (BEOL) integration. This work shows that the same stack supports low‐temperature non‐volatile storage and room‐temperature neuromorphic computing. More recently, Wan et al. achieved vdW integration of single‐crystalline‐quality BFO films with MoS_2_, preserving a clean interface that lowers the coercive field for polarization switching and improves subthreshold and read–write energy metrics [181]. Two FeFET architectures were demonstrated: a metal–ferroelectric–semiconductor (MFS) device with volatile memory and a metal–ferroelectric–metal–insulator–semiconductor (MFMIS) device with non‐volatile memory. Both operate at ultralow power (∼1.5 fJ·bit^−^^1^·µm^−^^2^ for MFS and 11.2 fJ·bit^−^^1^·µm^−^^2^ for MFMIS), offering an efficient route to low‐power memory and computing systems.

Oxide/TMD stacks illustrate how vdW assembly transfers oxide functionalities into low‐dimensional semiconductors. A complementary direction focuses on oxide/oxide heterostructures. These systems provide atomically sharp interfaces that can drive interfacial reconstruction and couple charge, spin, orbital, and lattice degrees of freedom beyond what is available in the bulk constituents. Twist engineering adds a programmable registry and symmetry degree of freedom within oxide/oxide junctions. It can imprint moiré‐like strain fields and strain gradients at the interface, which provides a direct route to engineer polar textures and their functionalities. One representative demonstration is that freestanding BTO membranes are stacked into homobilayers with programmable angles [45]. Without substrate clamping, depth‐resolved STEM–HAADF and 4D‐STEM directly reveal the coupling among twist‐induced moiré patterns, shear fields, and polarization textures. Specifically, at the maximum strain site between *AA* and *AB* registries, pronounced symmetric shear and antisymmetric rotation emerge. In a 15 nm, 11° sample, the shear at antivortex cores reaches ∼2.8%, but falls to ∼1% at 30 nm, indicating that thicker membranes dilute the modulation. Consistently, the toroidal moment drops from ∼4 to ∼1.5 e·Å^2^. Moreover, across twist angles (e.g., 3°, 10.4°), the vortex–antivortex lattice period tracks the moiré period and is thus set by the twist. The rotating polar textures are attributed to the flexoelectric coupling between polarization and the lateral, periodic shear/rotation fields at the interface. First‐principles calculations verified that this combined symmetric/antisymmetric shear and the 2D vortex modulation is in a low‐energy, stable state.

#### 5.2.3 Mechanical Oxide Heterostructures: Oxide/Graphene

**FIGURE 12:**
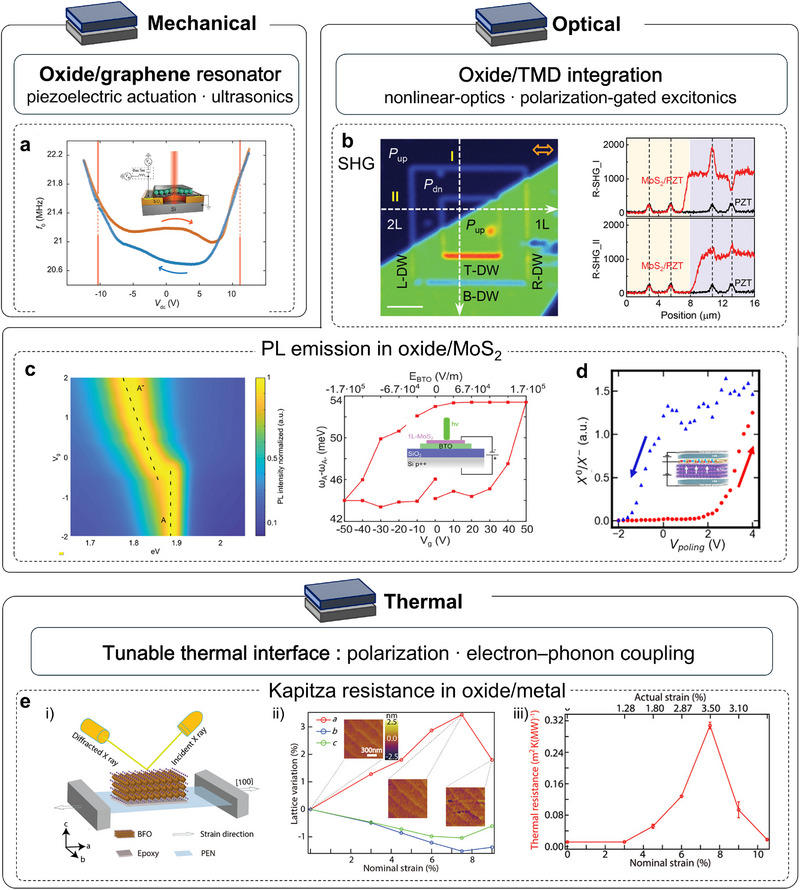
Mechanical, Optical and thermal properties of oxide heterostructures. (a) Resonance frequency *f*_0_ during a *V*_DC_ sweep [182]; inset, illustration of the experimental and device configuration. (b) Reflection SHG map and corresponding intensity profiles of the MoS_2_/PZT heterostructure with incident polarization indicated by open arrows; scale bar, 3 µm. R‐SHG intensity profiles for MoS_2_/PZT and bare PZT along the vertical (top) and horizontal (bottom) arrows [51]. (c) Peak photoluminescence (PL) mapping and gate‐bias dependence of the trion binding‐energy peak; inset, schematic of the dielectric device [183]. (d) Poling‐voltage dependence of the *X* ^0^/*X* ^−^ response showing hysteresis [184]; inset, schematic of the BTO/MoS_2_ dual‐gate device. (e) (i) Schematic of uniaxial stretching along the crystallographic *a* axis. (ii) Lattice evolution of a transferred BFO film (5.2 nm) under nominal strain: expansion along the stretch direction and contraction along the two transverse axes; nominal strain is defined as the percent elongation of the polyethylene naphthalate (PEN) substrate. (iii) Thermal resistance of the Al/BFO interface versus strain, showing a sharp increase with strain and a peak before lattice relaxation due to microcrack formation [185]. Panel c reproduced with permission: Copyright 2022, Wiley. Panel d reproduced with permission: Copyright 2024, American Chemical Society. Panel e reproduced with permission: Copyright 2021, Wiley.

Conventional electrostatic tuning of resonators relies on establishing a field between a suspended membrane and a bottom electrode to modify the membrane's vibrational modes. By contrast, freestanding oxide membranes readily form heterostructures with ultrathin, and even monolayer, 2D materials while retaining their large deformability, opening a route to in situ modulation of the oxide's intrinsic properties and, in turn, its mechanics. As a representative example, Lee et al. fabricated a suspended graphene/BTO/graphene heterostructure capacitor and drove it piezoelectrically [182] (Figure 12a). They used picosecond ultrasonics to resolve a bulk acoustic mode near 233 GHz, squarely within prospective 6G bands. This work elegantly couples correlated oxide piezoelectricity with graphene's mechanical and electrical advantages, providing a template for embedding oxide functionalities directly into RF‐grade resonators.

#### 5.2.4 Optical Oxide Heterostructures: Oxide/TMD and Oxide/Si

Semiconducting TMD monolayers serve not only as transistor channels but also as platforms with exceptionally strong nonlinear optical responses, second‐, third‐, and higher‐harmonic generation, positioning them for ultrafast light–matter studies and nanophotonic/optoelectronic devices. Freestanding ferroelectric oxides add a complementary control modality that augments strain and electrostatic gating while remaining compatible with vdW assembly. For example, Li et al. interfaced monolayer MoS_2_ with two PZT platforms [51] (Figure 12b) and via engineered ferroelectric domains and domain walls, reconfigurably modulated the second harmonic generation (SHG) amplitude and polarization of MoS_2_. Domain writing on the freestanding PZT affords in‐operando, non‐volatile, and scalable control of nanoscale nonlinear emission, enabling programmable, low‐voltage photonic functionality. To enhance and control the optoelectronic response of ferroelectric‐oxide/2D heterostructures, Pucher et al. assembled freestanding BTO with monolayer MoS_2_ and used polarization‐induced doping to precisely shift the exciton/trion balance [183] (Figure 12c). They achieved tunable trion binding energies of ∼40–100 meV with reversible PL modulation in a floating‐gate geometry exhibiting non‐volatile hysteresis. Meanwhile, freestanding ferroelectric BTO membranes were integrated with MoSe_2_ to realize a dual‐gate device [184]. Field‐controlled ferroelectric polarization in BTO modulates the PL of its excitons in MoSe_2_, verifying the charge transfer and coupling of the polarization and excitons at vdW heterointerface (Figure 12d). These works extend ferroelectric coupling from charge transport to exciton‐spectrum engineering, highlighting light–electric–ferroelectric three‐field coupling and the advantage of BTO's high‐*κ* polarization for low‐voltage optical control.

Freestanding STO membranes already host highly confined, low‐loss far‐infrared surface phonon polaritons, and engineered heterostructures extend this capability into Si‐integrated photonic architectures that are directly relevant to scalable on‐chip systems. Recently, Tao et al. laminated single‐crystal BTO (∼150 nm) onto silicon‐on‐insulator Mach–Zehnder interferometers, where the Si waveguide provides a low‐loss routing and interferometric readout platform while thinning of the top SiO_2_ to ∼5 nm by chemical‐mechanical polishing to strengthen the evanescent overlap [186]. By rotating the membrane in‐plane to align the electro‐optic tensor, they realized electro‐optical modulation with *V*_π_ *L* as low as 1.67 V·cm. Compared with earlier bonded‐epitaxial film demonstrations [187], this freestanding‐to‐waveguide integration exploits the declamped ferroelectric and introduces a novel “rotation‐angle engineering” strategy, enabling compact, low‐voltage, and Si‐compatible modulators.

#### 5.2.5 Thermal Oxide Heterostructures: Oxide/Metal

While freestanding oxides already exhibit improved intrinsic heat transport compared with substrate‐clamped films, practical devices still require electrodes and heterostructure integration. In these regimes, interfacial thermal conductance often becomes the dominant factor. At the nanoscale, boundary scattering largely sets the thermal resistance; thus the transduction of heat between the metal's electrons and the insulator's phonons becomes the rate‐limiting process. Because electrons and phonons are the principal heat carriers in metals and insulators, respectively, efficient cross‐interface heat flow hinges on electron–phonon energy exchange. In ferroelectric oxides, broken inversion symmetry and polarization charges further reshape the interfacial potential landscape. Zang et al. reported a large, dynamically tunable change in heat transport across metal/ferroelectric interfaces by nearly an order of magnitude [185] (Figure 12e). By using uniaxial strain to reorient polarization, they showed that bound charges strongly modulate electron–phonon coupling, enhancing the interface Kapitza resistance.

#### 5.2.6 Magnetic Oxide Heterostructures: Oxide/TMD, Oxide/Oxide

**FIGURE 13:**
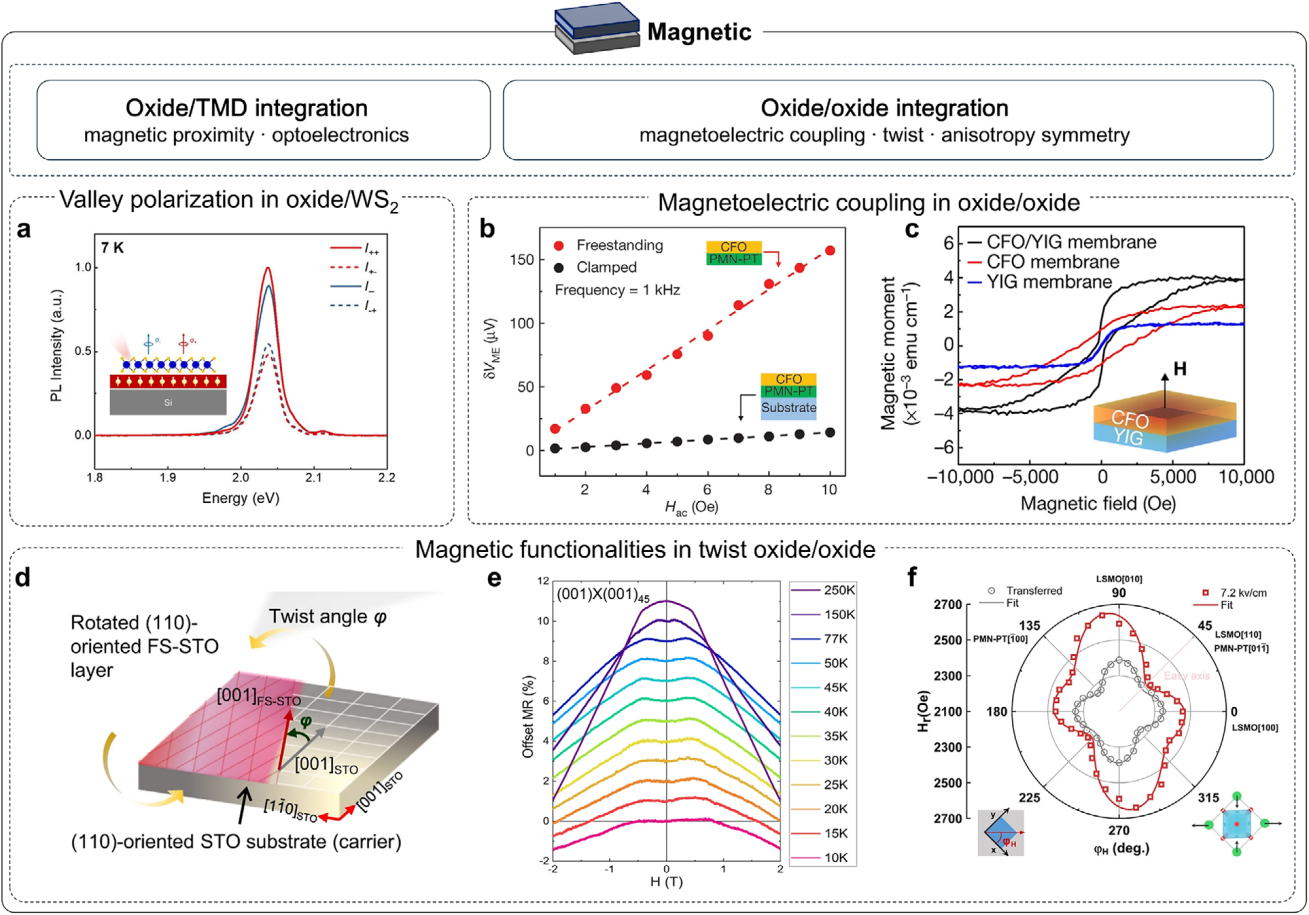
Magnetic properties of oxide heterostructures. (a) Circularly polarized PL from monolayer WS_2_ on freestanding LMO; inset, luminescence measurement geometry [188]. (b) Magnetoelectric voltage δ*V*_ME_ across PMN‐PT versus AC magnetic‐field amplitude at 1 kHz; inset, schematics of freestanding and clamped devices. (c) Out‐of‐plane magnetic hysteresis at room temperature for CFO membrane, YIG membrane, and CFO/YIG heterostructure [37]. (d) Schematic illustration of constructing an STO/STO homostructure with a designated twist angle. (e) Temperature‐dependent transport of (001)x(001) LSMO homostructures [190]. (f) In‐plane *H*_r_ anisotropy of transferred and stressed films at 7.2 kV cm^−1^, fitting values of *H*_u_ = 68 Oe and *H*_f_ = 55 Oe imply the coexistence of fourfold and uniaxial anisotropy in 45° sample [191]. Panels b and c reproduced with permission: Copyright 2020, Springer.

Beyond their widely used semiconducting character, TMDs exhibit strong spin–orbit coupling and spin–valley locking. Proximity exchange with an adjacent magnetic oxide can break the K/K′ valley degeneracy and make the magnetic order directly readable through circularly polarized optical responses or transport signatures. For example, Yang et al. integrated LSMO with MoS_2_ to realize multifunctional devices that combine dielectric gating with magnetic proximity exchange. In the LSMO/MoS_2_ stack (LSMO on top), a gate‐tunable rectifying junction operates as a metal–semiconductor FET. Reversing the order to MoS_2_/LSMO (MoS_2_ on top) yields a Schottky photodiode whose photoresponse and magnetic characteristics are tunable via the back‐gate voltage [39]. In addition, Lu et al. assembled a vdW bilayer of monolayer WS_2_ with a freestanding LMO membrane and showed that the magnetic oxide imprints valley polarization onto the TMD without growth‐induced disorder [188] (Figure 13a). The exchange‐proximity mechanism is direct. The large LMO moment lifts the *K*/*K*′ degeneracy in WS_2_, producing PL polarization that follows the LMO magnetic transition with temperature. In contrast to oxide–oxide stacks, this approach exports magnetic functionality into a valleytronic semiconductor while preserving a minimally bonded interface.

When magnetic oxides are integrated into freestanding heterostructures, neighboring layers can tune the oxide magnetism. The oxide can also strongly modulate the magnetic and electronic properties of adjacent layers. In 2020, Kum et al. stacked magnetic‐oxide heterostructures in the freestanding format [37] (Figure 13b,c). Two examples illustrate why declamping matters. In CFO/Y_3_Fe_5_O_12_ (CFO/YIG), the composite exhibits a sharper out‐of‐plane magnetization reversal than the arithmetic sum of the individual loops, attributable to residual in‐plane strain from thermal‐expansion mismatch that pivots the easy axis toward the normal and cleans the reversal pathway. In CFO/Pb(Mg_1/3_Nb_2/3_)O_3_‐PTO (CFO/PMN‐PT), the magnetoelectric response is enhanced by more than an order of magnitude relative to substrate‐clamped devices, because removing the substrate allows electric‐field‐induced strain in PMN‐PT to transmit fully and reversibly into the magnetic layer, strengthening magnetoelastic coupling. Later, Pesquera et al. transferred LSMO onto PMN‐PT, and quantified the declamped enhancement that electric‐field control of magnetization and anisotropy becomes markedly stronger [189]. This indicates that the absence of substrate constraint makes piezoelectric strain transfer more complete and energy‐efficient, thereby strengthening magnetoelastic coupling and lowering the fields needed to reorient magnetization.

In magnetic oxide/oxide junctions, the interfacial registry directly governs exchange pathways and the magnetic‐anisotropy energy landscape. Twist therefore, provides a continuous, programmable handle to reshape interfacial symmetry and the in‐plane strain tensor without changing composition. This enables deterministic control of anisotropy, magnetoelectric coupling, and low‐field transport. Freestanding oxide membranes introduce twist as a programmable interfacial degree of freedom for lateral homo‐ and heterostructures, creating atomically sharp in‐plane junctions whose geometry and symmetry can be set by rotation. The imposed angle reorganizes microstructure and interfacial symmetry, activating junction‐mediated transport pathways and reshaping anisotropy landscapes through angle‐dependent strain transfer and coupling. In this way, twist complements declamping to expand the accessible phase space, enabling functionalities that are difficult or impossible in conventional, substrate‐clamped epitaxy. For twist homostructure, Wu et al. transferred a freestanding (110) STO layer onto an (110) STO host so that covered and uncovered regions naturally form a tunable twist angle [190] (Figure 13d,e). LSMO grown atop this twisted template exhibits substantially enhanced low‐field magnetoresistance. The physics is attributed to the twist‐induced structural disorder that phase/grain boundaries and weak links create additional spin‐polarized tunneling barriers, thereby amplifying LFMR at small fields. Besides, Yang et al. extended twist control into magnetoelectric design by assembling LSMO/PMN‐PT with a defined interlayer twist [191] (Figure 13f). They observed the coexistence of fourfold and uniaxial magnetic anisotropies with a clear twist‐angle dependence. Twisting reshapes the in‐plane strain tensor delivered by PMN‐PT, which rewrites the magnetocrystalline‐anisotropy landscape in LSMO, showing twist as a deterministic handle on anisotropy symmetry and strength under electric‐field actuation, unifying angle–strain–polarization into a single, programmable space.

#### 5.2.7 Superconducting Oxide Heterostructures: Oxide/Oxide

Once monolayer high‐*T*_c_ superconductivity was established in BSCCO, it demonstrated that cuprate superconductivity can persist in the truly 2D limit, providing a natural platform for vdW twist homojunctions and heterointerface coupling studies. In 2021, Lee et al. used mechanical exfoliation and restacking to fabricate Bi‐2212/Bi‐2212 twisted vdW Josephson junctions and observed a pronounced twist‐angle dependence of the critical current, with maximum values near 0° and 90°, and a near‐vanishing value around 45°, consistent with d‐wave pairing and a finite “incoherent tunneling” contribution [192]. To achieve higher interfacial quality in twisted devices, end‐to‐end clean transfer, dual‐layer encapsulation, and low‐energy electrode deposition protocols have been introduced [193]. Subsequently, Wang et al. refined the clean transfer, encapsulation, and contact fabrication processes, and extended the study to Bi_2_Sr_2‐_ *_x_* La*_x_* CuO_6+_ *ᵧ* (Bi‐2201), a single–CuO_2_–layer cuprate. Their work further elucidated the angular dependence of interlayer Josephson coupling, highlighting how twist angle modulates superconducting coherence in the monolayer cuprate limit [194]. Later, Zhao et al. employed cryogenic assembly to realize atomically sharp, intrinsic‐quality interfaces in Bi‐2212 twisted junctions and uncovered time‐reversal‐symmetry–breaking superconductivity near ∼45°, evidenced by fractional Shapiro steps and reversible switching between two time‐reversal symmetry‐related ground states [47]. This programmable, magnet‐free Josephson diode behavior establishes the twist angle from a spectroscopic probe to a functional device parameter.

## 6 Outlook

The rapid advances in oxide membrane fabrication, precision lift‐off, and deterministic transfer have unlocked the broad opportunities of freestanding oxides. These developments have enabled the establishment of a comprehensive library of intrinsic properties, flexible integration for strain‐tunable functionalities, and the construction of vdW‐engineered heterostructures that exploit interfacial and proximity couplings, together propelling major progress in both fundamental science and device concepts (Table 2). Across these architectures, freestanding oxides exhibit a rich spectrum of physical properties, providing a robust foundation for diverse functional devices. Looking ahead, the evolution of this field will continue to follow these three complementary pathways, yet realizing the next generation of multifunctional devices will require overcoming several critical technical challenges as follows.

- Membrane quality: Despite progress toward better oxygen control and gentler growth/transfer, most freestanding oxides remain constrained by narrow growth windows and insufficient oxygen supply. Contamination of 2D spacers or sacrificial layers, together with pinholes and polymer residues, commonly yields rough oxide surfaces that preclude atomically conformal contact, especially with 2D materials, severely degrading heterostructure performance. Elemental and oxygen loss during transfer and annealing further amplifies oxygen vacancies and off‐stoichiometry, directly impairing electronic, magnetic, dielectric, and thermal transport. Closing these gaps will require an end‐to‐end process that preserves stoichiometry and surface integrity from growth through lift‐off, transfer, and post‐treatment. Operando readouts of oxygen chemistry can define processing windows that remain stable after encapsulation, annealing, and electrical bias. Progress should be assessed using wafer‐level thickness and roughness maps, statistically quantified defect densities, and distributions of key device metrics so that membrane quality reflects reproducible manufacturability rather than isolated best‐case samples.
- Wafer‐scale fabrication and transfer: Despite progress in inch‐scale remote epitaxy, stoichiometry‐optimized SAO sacrificial layers, and proposed single‐ and two‐step transfer schemes, the overarching goal remains a uniform, wafer‐scale, and stable oxide membrane platform. In practice, wafer‐level nonuniformity, interfacial ion depletion, and transfer‐induced cracking or wrinkling remain common, indicating that key process variables are not yet under feedback‐controlled, end‐point‐defined control. Scaling to production‐grade membranes therefore, calls for end‐point‐controlled lift‐off and transfer with in‐line metrology, paired with automation‐compatible handling that maximizes yield while minimizing contamination and mechanical damage. These shortcomings severely hinder the fabrication of flexible devices and the implementation of vdW/remote‐epitaxy heterointerface engineering, and represent critical, pre‐industrial bottlenecks for any credible pathway to manufacturing. Process maturity can be evaluated through wafer‐scale yield and uniformity maps, batch‐level distributions of key device metrics, and reliability under bending and thermal cycling.
- Heterostructure construction: Heterostructures are the key step that moves freestanding oxides from material demonstrations to application‐ready platforms. Current routes include oxide/oxide stacks spanning homo‐ and heteroepitaxial junctions, and oxide/2D assemblies enabled by clean vdW integration. In oxide/oxide systems, twist angle has been shown to tune interfacial reconstruction and electronic tuning. Progress toward small‐angle moiré engineering will rely on precise twist control and clean stacking to minimize contamination and angular uncertainty, together with systematic mapping across composition, thickness, and strain. Oxide/2D coupling offers a broader opportunity. Two‐dimensional materials span a wide band‐gap range, and can host correlated, ferroelectric, ferromagnetic, and superconducting states, yet most demonstrations exploit only a narrow subset. Broadening the accessible palette requires modular interface design through interlayers, encapsulation, contacts, and patterning that minimize interfacial trap formation and uncontrolled charge transfer while setting coupling strength in a controlled and reproducible manner.
- Multiphysics coupling: The central advantage of oxides lies in strong cross‐coupling among order parameters rather than simple coexistence. Treating any response as a standalone feature leaves performance unrealized and narrows the overall design space. Even early demonstrations that combine mechanics and ferroelectricity with thermal or optical functionality have already improved thermal and optoelectronic figures of merit [86]. This gain largely reflects the strong link between deformation and polarization. The next stage is system‐level co‐design of actuation and readout rather than post‐integration add‐ons. The coupling palette can then extend to magnetism, spin–orbit effects, and topological responses, with superconducting or electrochemical degrees of freedom introduced when appropriate. Freestanding boundary conditions broaden accessible symmetry and strain states. They can reshape electronic structure and interfacial reconstruction, making multi‐way couplings more attainable. Coupling‐enabled concepts must also remain stable in operation. Bending endurance, thermal cycling, and bias‐stress stability, therefore, become primary benchmarks.
- Si‐compatibility: The Si manufacturing platform underpins modern semiconductor technology. Integrating freestanding oxides into Si‐compatible process flows, therefore, provides a direct route from laboratory advances to deployment. Thus far, many demonstrations have centered on photonic waveguide coupling [105]. These studies exploit optical functionality while leaving much of the ferroic, electronic, magnetic, and thermal design space underexplored. Progress now hinges on converting isolated demonstrations into wafer‐relevant process flows, where bonding, transfer, and patterning operate within strict contamination and thermal budgets while maintaining interfacial chemical stability and low‐disorder electrostatics. This shift requires full‐stack integration beyond physical attachment, including process‐compatible encapsulation, contact formation, and back‐end routing, so that oxide ferroic and magnetic degrees of freedom can be co‐integrated with Si electronics and photonics on the same chip.

**TABLE 2:** Freestanding oxide membrane pathways and key advantages.

Development pathways	Key advantages	Representative examples
Strain‐free membranes	Maintained crystallinity at the ultrathin limit	**STO**: High‐quality membranes down to a single uc [43]
	Intrinsic response without epitaxial clamping	**STO**: anomalous, non‐monotonic thickness‐dependent bending modulus [84]
		**BFO**: switching voltage ∼40% lower and switching ∼60% faster [99]
		**SRO**: ferromagnetism enhancement (ΔT_c_ ≈ 10 K, ∼20% M_s_ increase) [116]
		**BFO**: BPVE photocurrent enhanced by ≈200% [104]
	Tunable boundary conditions	**STO**: wafer‐scale, arrayable trampoline resonators [87]
		**BTO**: environment‐sensitive phase window and domain topology [101]
		SRO: anneal‐driven self‐sealing for ultra‐hermetic cavities [88]
	Multiphysics coupling	**BTO**: photostrictive actuation in suspended nanodrums [86] **BTO**: UV‐induced mechanical displacement of 52 µm in suspended sheets [195]
Strained membranes	Superelastic compliance	**BTO**: near‐180° folding without fracture [44]
		**BFO**: ∼5.4% reversible bending and 180° folding [138]
	Mechanical tunability	**STO**: strain‐induced ferroelectricity under 2.0% uniaxial tensile strain [48]
		**STO**: wrinkle‐induced strain gradients yielding polar stripes [148]
		**LCMO**: switched from ferromagnetic metal to insulating antiferromagnet [49]
	Multiphysics coupling	**PTO**: SHG mapping of ferroelectricity under coupled strain–temperature tuning [144]
		**BTO**: deformation‐induced topological polarization for nonlinear beam shaping [52]
vdW‐integrated heterostructures	Atomically clean, sharp interface	**STO/MoS_2_**: vdW gap with leakage <10^−^^6^ A·cm^−^^2^ and robust electrostatic control [171]
		**BFO/MoS_2_**: reduced coercive field enabling ultralow‐energy FeFETs [181]
		**LMO/WS_2_**: valley polarization via exchange proximity without disorder broadening [188]
	Broad‐platform compatibility	**BTO/Si**: in‐plane polarization enabling rewritable domain‐wall memory [176]
		**Graphene/BTO/graphene**: piezoelectrically driven suspended capacitor [182]
		**BTO/SOI**: Mach–Zehnder with SiO_2_ thinned to ∼5 nm for stronger evanescent coupling [186]
	Multiphysics coupling	**Al/BFO**: strain‐tuned polarization boosting interfacial thermal resistance by ∼10× [185]
		**BTO/MoSe_2_**: polarization‐tuned exciton photoluminescence [184]

In conclusion, freestanding oxide membranes remove epitaxial clamping and establish an intrinsic, strain‐free baseline that can be programmed through engineered strain states, strain gradients, and external fields. This control is readily extended through vdW assembly, which enables heterointegration with Si and layered 2D materials. This platform supports coupled mechanical, dielectric, ferroelectric, magnetic, optical, thermal, and superconducting responses within a unified materials and device framework. Moving toward scalable technologies will require reproducible surface and interface quality, wafer‐scale lift‐off and transfer with high yield, and Si‐compatible integration within tight thermal and contamination limits. Resolving these bottlenecks will support robust translation from laboratory demonstrations to scalable, multifunctional oxide device systems.

## Conflicts of Interest

The authors declare no conflicts of interest.

## Data Availability

The authors have nothing to report.
